# Novel Oxidative Stress Biomarkers with Risk Prognosis Values in Heart Failure

**DOI:** 10.3390/biomedicines11030917

**Published:** 2023-03-15

**Authors:** Mei Li Ng, Xu Ang, Kwan Yi Yap, Jun Jie Ng, Eugene Chen Howe Goh, Benjamin Bing Jie Khoo, Arthur Mark Richards, Chester Lee Drum

**Affiliations:** 1 Department of Medicine, Yong Loo Lin School of Medicine, National University of Singapore, Singapore 119228, Singapore; 2 Vascular Surgery, Department of Cardiac, Thoracic and Vascular Surgery, National University Heart Centre, Singapore 119074, Singapore; 3 Department of Surgery, Yong Loo Lin School of Medicine, National University of Singapore, Singapore 119228, Singapore; 4 Cardiovascular Research Institute, Department of Medicine, Yong Loo Lin School of Medicine, National University of Singapore, 1E Kent Ridge Road, NUHS Tower Block, Level 9, NUHCS, Singapore 119228, Singapore; 5 Department of Biochemistry, Yong Loo Lin School of Medicine, National University of Singapore, Singapore 117599, Singapore

**Keywords:** oxidative stress, novel markers, heart failure, risk assessment, prognosis, surrogate markers

## Abstract

Oxidative stress (OS) is mediated by reactive oxygen species (ROS), which in cardiovascular and other disease states, damage DNA, lipids, proteins, other cellular and extra-cellular components. OS is both initiated by, and triggers inflammation, cardiomyocyte apoptosis, matrix remodeling, myocardial fibrosis, and neurohumoral activation. These have been linked to the development of heart failure (HF). Circulating biomarkers generated by OS offer potential utility in patient management and therapeutic targeting. Novel OS-related biomarkers such as NADPH oxidases (sNox2-dp, Nrf2), advanced glycation end-products (AGE), and myeloperoxidase (MPO), are signaling molecules reflecting pathobiological changes in HF. This review aims to evaluate current OS-related biomarkers and their associations with clinical outcomes and to highlight those with greatest promise in diagnosis, risk stratification and therapeutic targeting in HF.

## 1. Introduction

Heart failure (HF) is a global health problem, with an estimated global prevalence of 64.3 million people in 2017 [1]. Although mortality from cardiovascular diseases (CVD) has declined, the prevalence of HF continues to increase. In the United States alone, the prevalence of HF from 2015–2018 in those over 20 years of age was projected to increase by 3% from 6 to 8 million by 2030 [2,3]. In the UK, HF increased by 23% from 2002 to 2014, affecting 1.4% of the population [4]. In Southeast Asia, HF was higher at an estimated 4.5% to 6.7% [5]. This increase in HF prevalence is due to an ageing population, improved survival after myocardial infarction, poor adherence to HF prevention strategies, and increasing prevalence of cardiovascular risk factors [6]. The global burden of HF will continue to rise, leading to escalating healthcare expenditure. In 2012, the overall economic cost associated with HF globally was estimated to be USD 108 billion per annum, with most costs related to inpatient hospitalization [7].

Guidelines presently define HF as a chronic, progressive condition caused by any structural or functional impairment of ventricular filling or blood ejection [8]. HF consists of complex clinical signs and symptoms of shortness of breath (SOB), malaise, limb swelling or reduced exertional capacity caused by the failure of the heart to keep up with demands of the body [9]. Ejection fraction, or how much blood pumped by the heart with each beat, is one way to categorize HF. HF with reduced ejection fraction (HFrEF) is characterized by systolic failure caused by the left ventricle’s inability to contract normally (left ventricular ejection fraction, LVEF ≥ 50%). HF with preserved ejection fraction (HFpEF) is diastolic failure caused by the left ventricle’s loss of normal relaxation ability (LVEF < 40%) and the muscle has become stiff. HF with EF between 40 and 49% is defined as mid-range or mildly reduced EF (HFmrEF) LVEF between 40 and 49%.

HF can also be classified based on ACC/AHA staging criteria. There are four stages: Stage A—individuals at high risk for HF but without structural heart disease or symptomatic HF, Stage B—individuals with structural heart disease but without symptomatic HF, Stage C—individuals with structural heart disease with prior or current symptomatic HF and Stage D—refractory HF requiring treatment [8,9].

The development of pharmacotherapeutic agents with proven benefit in the treatment of patients with HFrEF has been progressing rapidly. Guidelines recommend stepwise initiation and up titration of ACE-I, ARBs and beta-blockers, and other pharmacotherapeutics such as mineralocorticoid receptor antagonists, angiotensin receptor neprilysin inhibitors and hyperpolarization activated cyclic nucleotide-gated channel blockers may be added [10,11]. Novel agents such as dapagliflozin and omecamtiv mecarbil have shown benefit in reducing mortality in patients with HFpEF [12,13], but improvements in patient outcomes and mortality have not been replicated. Multiple trials are currently underway to evaluate the effects of these agents on both cardiac structure and function and upon hard clinical end points in HFpEF [14].

Despite advances in HF management, re-hospitalizations and mortality associated with HF remain [15,16]. We need improved tools to aid in diagnosis, prognostication, and treatment. A HF diagnosis is made through presentation of symptoms in a patient. Further tests are conducted in the form of imaging techniques such as an electrocardiogram (ECG) or an echocardiogram (ECHO). These have been useful in the assessment of HF in patients [17,18,19,20,21,22,23,24,25,26]. The gold-standard for detecting HF is through a simple blood test looking for NT-pro-brain natriuretic peptide (NT-proBNP) and its active form brain type natriuretic peptide (BNP). Since its discovery in 1988, these markers are now recommended in guidelines such as the European Society of Cardiology (ESC) Clinical Practice Guidelines and the American (AHA/ACC/HFSA) Guidelines for the assessment of HF [27,28,29,30,31]. Nevertheless, this marker is already more than 30 years old, and a review is long overdue to ensure relevance in diagnostic and prognostic tools for HF.

Oxidative stress (OS) has been shown in multiple studies to be strongly implicated in the pathogenesis of HF. In this article, we provide a thorough review and discuss the role of OS in development and progression of HF. We also highlight selected OS biomarkers of high potential clinical relevance in HF.

## 2. Mechanisms of Oxidative Stress in Heart Failure

### 2.1. Overview of ROS and RNS

OS is caused by excessive production of Reactive Oxygen Species (ROS) and Reactive Nitrogen Species (RNS), two major redox biomarkers. Sies first defined it as a disturbance in the prooxidant-antioxidant balance in 1985 [32]. ROS act as intermediates in cellular pathways and can be classified into either free radicals such as superoxide (O_2_^−^) or hydroxyl (·OH) or non-free radicals such as hydrogen peroxide (H_2_O_2_) and peroxynitrite (ONOO^−^). Reactive nitrogen species (RNS) are species derived from nitric oxide and superoxide through the enzymatic activity of inducible nitric oxide synthase 2 (NOS_2_) and NOX [32].

### 2.2. Normal Physiology of ROS and RNS

Normally, ROS/RNS are generated in small amounts in the heart by enzymes such as NADPH oxidases, nitric oxide synthase (NOS), xanthine oxidase (XO) and mitochondria and are play a pivotal role in modulating cell cycle homeostasis, excitation–contraction coupling and cell function. NAPDH oxidases are transmembrane enzymes that specialize in producing O_2_^−^ from molecular oxygen by transfer of an unpaired electron from NADPH. The major isoform of NOX in the heart is NOX4, which is expressed primarily in the mitochondria of cardiac myocytes [33]. Inhibition of XO with allopurinol or oxypurinol in animal HF models led to protection of myocardial contractility, improved left ventricular function and the reversal of maladaptive cardiac remodeling [32].

NOS is responsible for endogenous production of nitric oxide (NO), an important signaling molecule in humans. When tetrahydrobiopterin (BH4) is sufficient, L-arginine and O_2_ are catalyzed by NOS to become l-citrulline and NO. However, in pathological conditions, uncoupling of NOS occurs and O_2_^−^ is generated as a by-product of NO synthesis during electron leakage at the electron transport chains during mitochondrial respiration. Complexes I and III of the electron transport chain are major sites of ROS generation, but under pathological conditions, the ROS from mitochondria is markedly increased.

### 2.3. ROS/RNS and the Induction of Cardiomyopathy

Cardiac hypertrophy and maladaptive remodeling are common in patients with HF, with progressive cardiomyocyte hypertrophy contributing to the development of HF. ROS/RNS is implicated in the downstream mechanistic underpinning of cardiomyocyte hypertrophy due to RAAS and sympathetic nervous system activation. Yasunari et al. demonstrated a significant correlation between left ventricular mass index and ROS/RNS levels in 104 hypertensive patients with left ventricular hypertrophy [34]. Hirotani et al. added that ROS/RNS is involved in the activation of NF-κB by G-protein-coupled receptor agonists such as Ang II or ET-1 [35]. Animal studies have also shown a close link between ROS and cardiomyocyte hypertrophy, which can be slowed by antioxidants.

An important pathway, Ang II induces cardiac hypertrophy by activating angiotensin II (AT1R) receptor, which induces ROS/RNS generation and activation of multiple downstream signaling kinases. This promotes cardiomyocyte survival and suppression of apoptosis. The c-Rel subunit of NF-κB has also been identified as a major promoter of cardiac hypertrophy in mice. Ang II stimulation of the Nox4-histone deacetylase (HDAC) axis leads to increased ROS production and nuclear export of HDAC. HDAC suppresses pro-hypertrophic transcription factors, but with HDAC loss, this suppressive effect is lost.

### 2.4. ROS/RNS and Cardiac Fibrosis

**Figure 1:**
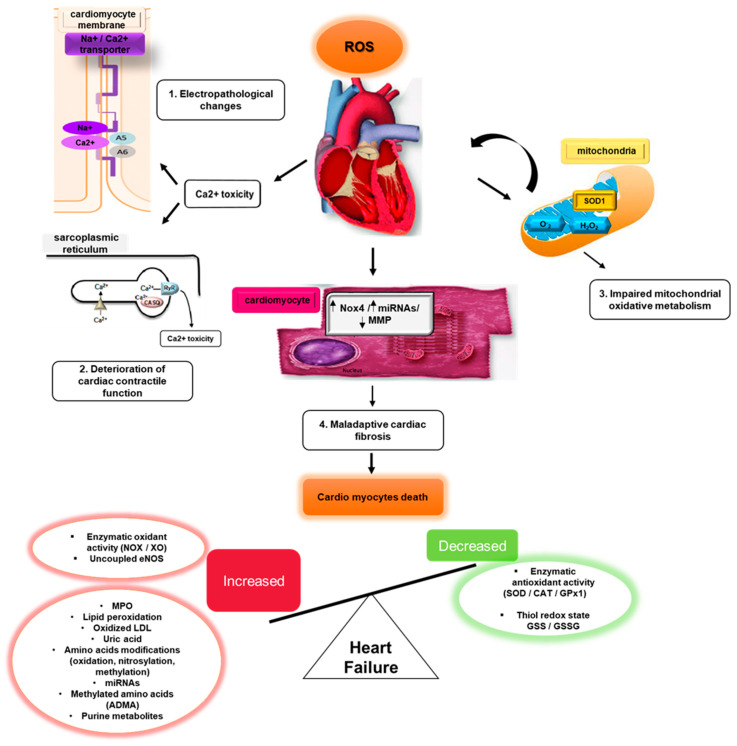
Mechanisms and expression of OS biomarkers in heart failure. Excessive ROS/RNS leads to cardiomyocyte hypertrophy and cardiac fibrosis in heart failure.

Cardiac fibrosis is a major pathological mechanism in HF (Figure 1), with increased extracellular matrix deposition from cardiac fibroblasts transitioning to secretory myofibroblasts. ROS and RNS mediate cardiac fibrosis, and Ang II upregulates transforming growth factor-β1 (TGF-β1) expression by activating AT1R and NOX4.

Matrix metalloproteinases (MMPs) are known to be pathologically activated by ROS through binding and opening of active sites on MMPs. This prevents TIMPs (Tissue Inhibitors of MMPs) from accessing the active site [36], ultimately ceasing activity and causing the onset of cardiac fibrosis. MMPs also regulate extracellular matrix remodeling, and alteration can lead to abnormal deposition and eventually dilated cardiomyopathy [30]. Left ventricular remodeling and HF onset is also thought to have been caused by MMPs [37,38,39], and have been associated with increased left ventricular internal dimensions and wall thickness [40]. This suggests that MMP may be a mediator and possible marker of cardiac remodeling and can be regulated by various ROS-related pathways, such as Ang-II induction of NF-κB and AP-1 transcription factors [41]. Aldosterone, part of the renin–angiotensin–aldosterone cascade (RAAS) commonly activated in HF, has altered levels of MMPs in animal studies. ROS/RNS has also been shown to affect MMPs directly by protein modification. These findings have been validated in several in vivo and in vitro studies [37,38,39].

### 2.5. Oxidative Stress in the Right Ventricle

The right ventricle (RV) is highly susceptible to OS compared to the left ventricle (LV) due to its inability to regulate expression of manganese superoxide dismutase, a key enzyme that attenuates ROS [42]. A high degree of OS adversely affects pulmonary vasculature and induces RV remodeling [43]. Pulmonary vascular remodeling due to OS may lead to hypertrophy and HF.

A key condition of OS-mediated HF is pulmonary hypertension (PH). During PH development, ROS generation increases due to monocytes accumulating in pulmonary arterioles [44]. An animal experimental model by Khoo et al. [45] revealed high radical formation in high-fat diets of rats. Investigating the importance of the balance between antioxidants and oxidants, Zelko et al. [46] knocked out SOD3 in mice and induced PH using silica. The resulting mice exhibited much higher RV pressures compared to the wildtype.

## 3. Redox Biomarkers in Heart Failure

### 3.1. Classical Biomarkers

While it may be intuitive to determine HF severity with ROS, there are several reasons that make ROS poor biomarkers to measure directly. Firstly, ROS have short half-lives and are not stable enough for sample collection and storage. Secondly, current methods of measurement such as spin trapping-based electron paramagnetic resonance spectroscopy are difficult. Thirdly, ROS are found in specific compartments in the body, which may render them inaccessible and difficult to quantify. Fourthly, it may be difficult to obtain adequate tissue samples as measurement is difficult. Despite multiple ways to measure and quantify oxidative states, no method is superior. As there are no suitable method for direct measurement, this has not yet entered clinical practice.

**Figure 2:**
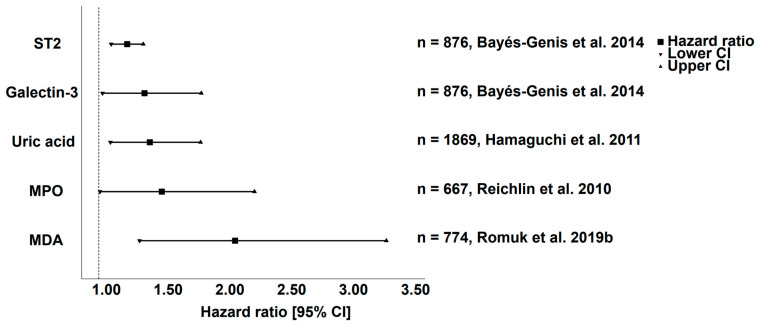
Oxidative stress-related biomarkers on all-cause mortality in heart failure [48,62,67,76].

Indirect markers of OS are more readily measured than direct assay of ROS, but use of surrogate markers is usually at the expense of differentiating specific ROS involvement. Most studies of OS and HF have used surrogate markers due to feasibility and better correlation to indices of HF severity. An ideal biomarker for HF would be one that is stable enough to be stored, has good assay reproducibility, sensitivity, specificity, is integral to the pathogenesis of disease and has rigorous evidence supporting its application in guiding disease management and improving important clinical outcomes. Table 1 summarizes promising OS biomarkers with potential clinical relevance, while Table 2 summarizes surrogate biomarkers with potential clinical relevance. Figure 2 depicts representative studies of the prognostic value of OS biomarkers highlighting malondialdehyde (MDA), myeloperoxidase (MPO), nitrotyrosine and uric acid (UA) as key prognostic markers on all-cause mortality in HF. Ghezzi et al. suggested a classification of OS biomarkers to clearly categorize biomarkers in their relation to OS. Briefly, there are 5 categories which biomarkers may fall into—ROS species themselves, oxidized products of OS, indicators of biochemical pathways associated with OS, antioxidant capacity and genetic biomarkers. It should be noted that these categories are not mutually exclusive [47].

**Table 1:** Oxidative stress-related biomarkers and clinical outcomes (up to 5 years) in heart failure.

Oxidative Stress-Related Biomarkers	Prognostic Value			Study Information		
	Prognostic Indicator	Reported Value (95% CI)	*p*	Endpoint	Sample (*n*)	Ref.
MDA	HR	2.103 (1.330–3.325);	<0.01	ACM	774	[48]
	RR	MDA 4th quartile vs. 1st quartile: 3.64 (1.917–6.926)	<0.01	ACM		
	HR	2.000 (1.366–2.928)	<0.001	Death, Heart transplant		
	R-value	ICM −0.111; nICM 0.152	<0.05	Severity (NYHA class)		
	HR	3.33 (1.55–7.12)	0.002	ACM	189	[49]
	HR	2.202 (1.296–3.741)	0.004	CVM, Heart transplant	707	[50]
Bilirubin	HR	1.47 (1.19–1.82)	0.0004	Pump failure death	1135	[51]
	HR	1.052 (1.001–1.099)	0.034	ACM, Hosp	556	[52]
OxLDL	HR	1.013 (1.003–1.024)	0.013	Mortality, Hosp	284	[53]
	HR	46.6 (1.5–1438.1)	0.02	Death, VAD, Heart transplant	60	[54]
F2-isoPs	OR	1.8 (1.1–3.1)	<0.05	CVM	392	[55]
	*p*-value	Symptomatic HF 0.0003; Left ventricular end-diastolic diameters 0.008; Left ventricular end-systolic diameters 0.026	<0.05	Severity (NYHA class)	51	[56]
	AUC	0.99	<0.001	Hosp, Severity	73	[57]
Protein carbonyls	AUC	0.84 (0.77–0.92)	<0.001	Severity (Prognosis)	104	[58]
8-OHdG	R-value	LVEF −0.27; PCWP 0.31; LVEDV index 0.22	0.0006; 0.0136; 0.0077	Severity	225	[59]
	R-value	Left atrial diameter 0.54; LVEDD 0.65; LVESD 0.66	0.002; 0.0001; <0.0001	Severity	46	[60]
	HR	2.02 (1.01–4.03)	0.0473	CVM, Hosp	272	[61]
UA	HR	ACM 1.413 (1.094–1.824); CVM 1.399 (1.020–1.920)	0.008; 0.037	ACM, CVM	2675	[62]
	RR	History of gout 1.63 (1.48–1.80) Acute gout episode: 2.06 (1.39–3.06)	<0.001; <0.001	ACM, Hosp	25,090	[63]
	HR	ACM 1.35 (1.07–1.72); CO 1.32 (1.06–1.66)	0.010; 0.020	ACM, CO	1152	[64]
	HR	ACM 2.24 (1.49–3.37); CVM 1.14 (1.06–1.23); CO 1.26 (1.01–1.56)	<0.001	ACM, CVM, CO	NA	[65]
MPO	AUC	0.909	<0.0005	ACM	1360	[66]
	HR	1.51 (1.01–2.26)	0.045	ACM	667	[67]
	AUC	0.53	0.045	ACM		
	HR	3.35 (1.52–8.8)	0.002	ACM, Hosp, Heart transplant	140	[68]
AGE	HR	Soluble RAGE 1.90 (1.16–3.09); Pentosidine 1.59 (1.11–2.29)	0.010; 0.012	CVM, Hosp	160	[69]
	HR	CML (Hosp) 1.20 (1.05–1.37); CML (ACM) 1.24 (1.07–1.45); Pentosidine (Hosp) 1.15 (1.00–1.31)	0.001; 0.006	ACM, CO (ACM, Hosp), Hosp	580	[70]
Nitrotyrosine	*p*-value	Higher in NYHA class III: NYHA III vs. NYHA II < 0.05 NYHA III vs. NYHA I < 0.03 NYHA III vs. Control < 0.02	0.010	Severity (NYHA class)	66	[71]
	R-value	proBNP 0.32; MPO 0.37	<0.010; <0.003	Severity (NYHA class)		
ST2/Galectin-3	HR	Galectin-3: Pump failure 1.12 (1.01–1.23) SCD 1.10 (1.00–1.22) ST2: Pump failure 2.27 (1.48–3.49)	0.850	Pump failure, SCD	813	[72]
	OR	120 days Hosp 2.79 (1.75–4.44); 120 days CO 1.84 (1.19–2.86)	<0.001	CO (ACM, CVM, Hosp), Hosp	902	[73]
	HR	HF 0.24 (0.16–0.70); Control 0.14 (0.13–0.17)	<0.0001	Severity	240	[74]
	HR	1.9 (1.3–2.9)	0.002	CO (Heart transplant, ACM)	1141	[75]
	AUC	0.75 (0.69–0.79)	0.240			
	HR	5-year ACM ST2 0.770 (0.746–0.793); 5-year CVM ST2 0.783 (0.753–0.813)	0.004; 0.040	ACM, CO, CVM	876	[76]

ACM—all cause mortality; AGE—advanced glycation end-products; AUC—area under curve; CAD—coronary artery disease; CI—confidence interval; CO—combined outcomes; CML—N-E-(carboxymethyl) lysine; CTnT—cardiac troponins; CVM—cardiovascular-associated mortality; EF—ejection fraction; F2-isoPs—F2-isoprostanes; HF—heart failure; Hosp—hospitalization; HR—hazard ratio; ICM—ischemic cardiomyopathy; IVCD—intraventricular conduction delays; KM curve—Kaplan Meier curves; LVEF—left ventricular ejection fraction; LVEDD—dilated left ventricle end diastolic volume; LVEDV—left ventricle end diastolic volume; LVESV—left ventricle end systolic volume; MACE—major adverse cardiovascular events; MDA—malondialdehyde; MPO—myeloperoxidase; NA—not applicable; NT-proBNP—NT-pro B-type natriuretic peptide; NYHA—New York Heart Association; OR—odds ratio; OxLDL—circulating oxidized LDL; *p*-value—probability value; PCWP—pulmonary capillary wedge pressure; 8-OHdG—deoxyguanosine; ST2—suppression of tumorigenicity 2; QTc—corrected QT interval; RAGE—receptor for advanced glycation end-products; R-value—Pearson’s correlation coefficient; RR—risk ratio; SCD—sudden cardiac death; Spearman’s rho—Spearman’s rank-order correlation coefficient; U-test—Mann–Whitney *U* test; UA—uric acid; VAD—ventricular assist device.

**Table 2:** Surrogate biomarkers associated with clinical outcomes (up to 5 years) in heart failure.

Surrogate Biomarkers	Prognostic Value			Study Information		
	Prognostic Indicator	Reported Value (95% CI)	*p*	Endpoint	Sample (*n*)	Ref.
SOD	C-Statistics	0.85 (0.77–0.92)	0.034	ACM, Hosp	593	[77]
	HR	1.07 (1.02–1.13)	0.005	ACM, Heart transplant	109	[78]
Thiol-containing compounds	Spearman’s rho	Cystine −0.31	0.007	Peak Filling Rate	75	[79]
	U-Test	GSH 21%	<0.0001	NYHA Class 1	91	[80]
PON1	KM curve	-	0.023	HF	299	[81]
	HR	Prognosis 2.63 (1.97–3.50); 3-year MACE risk 2.04 (1.49–2.79)	<0.01	MACE, CAD	3668	[82]
Ceruloplasmin	HR	1.90 (1.40–2.80)	<0.001	ACM	164	[83]
	*p*-value	Cardiomyopathy < 0.001; NYHA class < 0.05	<0.001; <0.05	Severity (NYHA class)	202	[84]
	HR	2.95 (1.29–6.75)	0.011	ACM	131	[85]

ACM—all cause mortality; CAD—coronary artery disease; CI—confidence interval; GSH—glutathione; HF—heart failure; Hosp—hospitalization; HR—hazard ratio; KM curve—Kaplan Meier curves; MACE—major adverse cardiovascular events; NYHA—New York Heart Association; PON1—paraoxonase-1; SOD—superoxide dismutase; Spearman’s rho—Spearman’s rank-order correlation coefficient; U-test—Mann–Whitney *U* test.

### 3.2. Biomarkers and Organ Specificity

Given the large number of possible biomarkers, it is difficult to decide which may prove most useful and where most effort should be directed into. The biomarkers of oxidative stress study (BOSS) studied 16 products of OS in the blood, plasma and urine of rats and found that MDA and isoprostanes in plasma and urine, and 8-OHdG in urine are potential candidates for general biomarkers of OS. As such, these markers have become the more widely studied.

Another reason why a specific biomarker may be selected is its organ-specificity. A biomarker becomes diagnostically superior if it points to a specific dysfunctional organ. There is a lack of study on the specificity of biomarkers in relation to diseased organ systems/tissues. Most studies focus on biomarkers from noninvasive plasma and urine samples to study a wide variety of pathologies. While some biomarkers show stronger correlation to pathologic states (such as isoprostanes in Alzheimer’s disease) [86], the diagnostic utility of OS biomarkers may be questioned, as they merely reflect an underlying state common to many diseases.

Furthermore, studies that use tissue samples often do not compare biomarkers different samples from various organ systems. Jun et al. found that intermittent hypoxia in mice lead to an increase in lipid peroxidation in the liver but not in heart or aortic tissues after 4 weeks. The effect was significant after 4 weeks, leading to a 38% increase in hepatic MDA levels. In other animal studies [87], suggested that vaccination in rainbow trout induced lipid peroxidation in gill and liver tissues but muscle and brain tissue can restore its pro- and antioxidant balance after vaccination.

### 3.3. Lipid Peroxidation

#### 3.3.1. Malondialdehyde

Malondialdehyde (MDA) is an organic compound that consists of a highly reactive dialdehyde with two carbonyl groups. It is generated as an end-product of lipid peroxidation of membrane polyunsaturated fatty acids (PUFAs) in response to the presence of free radicals and ROS [88]. In physiological conditions, MDA is relatively inert, but in low pH, it can form covalent adducts with amino acids, such as the Lys-MDA-Lys crosslink. MDA is easy to quantify spectrophotometrically using thiobarbituric acid reactive substances (TBARS) assay, but this test lacks specificity for MDA as other aldehydes can form products that absorb light in the same range as MDA. ELISA assays have shown good performance with increased specificity compared to using TBARS [88].

MDA is often used as a biomarker in OS studies and has generally been considered a by-product of, rather than contributory to HF development. However, Folden et al. found MDA to have a depressive effect on ventricular contractile function at a single cardiomyocyte level, by direct phosphorylation of P38 MAP kinase [89]. P38 has been associated with the onset of HF [90] and is known to have a negative inotropic effect, due to reduced responsiveness of myofilaments to Ca^2+^ [91]. However, a study found that it only correlated with HF when compared to healthy controls. There was no significant difference in symptomatic or asymptomatic groups, suggesting that MDA may be more sensitive and may pick up HF earlier in disease course [92]. However, another study suggested that there was graded elevation of MDA according to New York Heart Association (NYHA) classification, with Class II NYHA patients showing significantly lower MDA levels and significantly higher levels of vitamin A, vitamin E, lutein, and lycopene than Class III patients, and correlation between elevated MDA levels and poorer ejection fraction [93].

MDA has also been studied in the etiology of HF. A study evaluating a large group of 479 patients with ischemic HF and 295 patients with non-ischemic HF showed higher total antioxidant capacity, MDA concentration and MDA/PSH ratios in ischemic cardiomyopathy. Other parameters, including total oxidant status, UA, and albumin, measured in the study did not differ between the groups [94]. The results suggest MDA could be a useful marker to differentiate HF etiology.

MDA also shows potential as a prognostic marker for HF. Romuk et al. found higher MDA and UA to be independent predictors of death and the combined endpoint of death and heart transplantation, in patients with chronic HfrEF. Other markers of oxidative balance including total antioxidant capacity, total oxidant status, OS index and protein sulfhydryl groups were measured but were not found to be prognostically useful [48]. Radovanovic et al. and Wojciechowska et al. also found MDA to predict mortality in patients with chronic ischemic HF [49,50]. In uremic patients, levels of MDA and oxidized low-density lipoprotein (oldy) were elevated in those with HF compared to non-HF cases, suggesting that MDA may be used as a marker for HF in uremic patients [40].

Lipid peroxidation assessed by measurement of MDA and isoprostanes was surprisingly not increased in patients with left ventricular dysfunction treated with standard HF therapy. No correlation was found to the severity of HF, suggesting that the lack of elevation of markers of lipid peroxidation may reflect effective treatment of HF [95]. MDA remains a strong contender as a potential biomarker for HF, given its ability to suggest etiology and medical therapeutics.

#### 3.3.2. Pentane and Acetone

Pentane is an end-product of lipid peroxidation, produced from the reaction between free radicals and omega-six fatty acids and excreted through the lungs. Breath pentane excretion is a reliable noninvasive index of lipid oxidation, and patients with chronic heart failure (CHF) excreted significantly higher pentane concentrations than control subjects, regardless of whether they had been treated with captopril or enalapril [96]. A study was conducted to determine if the dose-dependent relationship between captopril and pentane exhalation was due to the sulfhydryl group, which was hypothesized to have radical scavenging capability. The study found that pentane was significantly increased in class IV compared to class II, but there was no significant difference in excretion between class III and either classes II or IV [97]. While excreted pentane shows potential as a diagnostic marker, it does not significantly correlate with NYHA functional class [96].

Exhaled breath acetone (EBA) is another volatile organic compound reported to be increased in HF patients due to an altered metabolism and shift from carbohydrates to fat as a source of energy. A study found that diabetic patients with more severe HF have elevated EBA. While EBA is also detected in patients with other diseases such as diabetes mellitus, lung cancer, and allergic asthma, it may still have potential in differentiating underlying HF [98]. EBA concentrations were also higher in more severe right sided-venous engorgement, suggesting that hepatic congestion may contribute to ketosis generation [99].

Patients with lower EBA levels and pentane upon initial hospital admission have been shown to have a significantly higher likelihood of survival [100]. A recent study of 695 patients with chronic HF concluded that higher EBA levels were independently correlated with cardiac and overall mortality over a follow-up period of 18 months [99,101]. With advances in metabolomics, EBA is indeed a promising biomarker with good correlation with HF severity and prognosis.

#### 3.3.3. Biopyrrins

Bilirubin generation is increased under OS due to the induction of heme oxygenase, an enzyme that catalyzes the degradation of heme and is rate-limiting in bilirubin synthesis. Bilirubin has antioxidant effects by scavenging ROS and is particularly beneficial against lipid peroxidation and LDL oxidation. It is subsequently oxidized to form biopyrrins, which can be measured using anti-bilirubin monoclonal antibodies in urine samples. An inverse relationship between serum bilirubins and coronary artery disease (CAD) has been established, suggesting that it may have a cardiovascular protective effect [100].

In HF studies, biopyrrin levels were significantly higher in patients with HF and correlated with NYHA functional classification. Log biopyrrin to creatinine levels correlates with other markers of cardiac function, including a positive correlation with pulmonary artery wedge pressure, pulmonary artery pressure and log BN; it has a negative correlation with the cardiac index and left ventricular ejection fraction. Standard medical therapy of HF was shown to reduce both biopyrrin levels and NYHA functional classification in parallel [101].

In human studies, biopyrrins have been used to measure OS in various cardiovascular pathologies, as well as reperfusion injury studies. In patients with myocardial infarction, urinary biopyrrin levels were elevated once corrected for serum creatinine and were significantly higher than in patients with stable angina. Following intervention, coronary reperfusion was associated with increased urinary biopyrrin, which trended as normal from 24 h to 7 days [102]. The authors concluded that OS may be contributory to the complication of reperfusion injury. In atrial fibrillation, biopyrrins levels were reduced following sinus rhythm restoration in persistent atrial fibrillation patients [103], suggesting that atrial fibrillation itself may be a source of OS.

There is lack of evidence regarding the use of biopyrrin as a prognostic marker for HF, and more studies are needed. Interestingly, bilirubin itself, while not a marker of OS, has been shown to be a prognostic marker for HF in multiple studies. The increase in bilirubin has been explained by back pressure of the failing right ventricle resulting in hepatic congestion and hepatocyte atrophy [104], although it may also be a response to OS in HF through the stress-induced activation of heme-oxygenase 1, which is responsible for oxidative cleavage of heme groups leading to generation of biliverdin and its anti-oxidative effects [105].

Most studies have found bilirubin to be associated with severity and poor prognosis. HF. Wu et al. found that increased bilirubin level was associated with decreased survival rates and the risk of pump failure death [51]. Okada et al. found direct bilirubin to predict all-cause mortality in acute decompensated HF [52], while Chintanaboina et al. found serum total bilirubin to be an independent risk predictor of hospital admissions secondary to HF [106]. However, Zheng et al. found that patients with HFpEF had lowered bilirubin compared to healthy controls, and increased severity of HFpEF was associated with significantly lower total bilirubin levels [107]. They postulated that the correlation could be explained by the reduced antioxidant capacity in decreased bilirubin levels for more severe HFpEF.

Since bilirubin is a precursor of the oxidized biopyrrin, it is reasonable to expect levels of bilirubin to correlate with biopyrrin. Indeed, studies which measured both metabolites have found bilirubin to correlate with biopyrrin in serum and urine [108,109]. Therefore, it may be reasonable to hypothesize that biopyrrins may confer a similar prognostic value. Future studies involving biopyrrins in conjunction with bilirubin may be able to help determine the major source of bilirubin production in HF and the mechanism linking bilirubin and HF, both for HFpEF and HFrEF.

#### 3.3.4. Oxidized LDL

ROS oxidizes LDL and phospholipids to form OxLDLs, which are taken up by macrophages and overlying endothelial cells to form foam cells and proinflammatory cytokines. These cytokines are mostly detected by circulating monoclonal antibodies which are specific for the different epitopes of OxLDL. OxLDL has good reproducibility and stability in storage.

OxLDL has been linked to HF due to its ability to induce poor Ca^2+^ handling and decreased paraoxonase 1 (PON1) activity [40,110]. It may also oxidize to form 7-ketone cholesterol (7KCh) which may induce OS in cardiomyocytes leading to cell death. Elevated OxLDL levels in HF correlate with decreased paraoxonase 1 (PON1) activity (further discussed separately below), suggesting that there is impairment of the antioxidant system and metabolism of OxLDL. It is also associated with NT-proBNP in young subjects with or without stable coronary artery disease [111]. High OxLDL levels have been linked to a more spherical left ventricular cavity [112], and decreasing diastolic and systolic function independent of other inflammatory markers, lifestyle or vascular cardiac structure [113,114]. OxLDL appears to be correlated to HF severity, with plasma OxLDL being significantly higher in severe CHF patients than in control subjects and mild CHF patients [115]. Several other studies have confirmed the correlation between OxLDL and HF severity.

Jorde et al. found that high plasma levels of BNP and OxLDL were independent predictors of mortality in HF patients, while other measured variables such as LVEF and neuro-hormonal factors did not [54]. After maximal exercise, patients post-exercise of more than 11.0 U/L with a higher increase in OxLDL had an increased risk of death and need for ventricular assist device or heart transplant. OxLDL antibodies (OxLDL Abs) are an alternative to direct measurement of Ox-LDL levels. They are the product of immune response to OxLDLs and express a variety of epitopes and induce the production of a polyclonal mixture of IgA and IgG antibodies, which can predict morbidity and mortality. However, lowering OxLDL with antioxidant therapies has not been shown to decrease rates of cardiovascular events. A study of 353 healthy subjects revealed that supplementation decreased circulating OxLDL but did not slow down the progression of carotid artery intima-media thickness over a 3-year period [116].

#### 3.3.5. F2-Isoprostanes

F2-isoprostanes (F2-isoPs) are a class of prostaglandin-like compound produced from the peroxidation of arachidonic acid derived from the cellular phospholipid bilayer in the presence of ROS or cellular OS. They are produced independent of cyclooxygenase enzyme and can only be detected in very low concentrations in biological fluids. They are one of the most reliable markers for monitoring OS due to their stability and sensitivity to OS exposure. They are detectable in both urine and serum samples, allowing easy and noninvasive sample collection, and concentrations in samples are independent of hepatic and renal function. Furthermore, concentrations in samples are independent of hepatic and renal function, making them viable biomarkers in patients with organ impairment. Currently, the best method of quantification involves gas chromatography or liquid chromatography with mass spectrometry [117]. Other simpler options include enzyme-linked immunosorbent assay (ELISA) and radioimmunoassay.

Isoprostanes are a biomarker of OS and may be contributary to CVD. They mediate vasoconstriction through potentiating the effects of noradrenaline and angiotensin [118], increasing endothelin expression, and vascular smooth muscle cell [119]. The mechanism of their effects was initially thought to involve thromboxane A2 (TxA2) receptors [120], but there is increasing evidence that there is a distinct receptor for isoprostanes. Fukunaga et al. showed isoprostanes displaces TxA2 agonists less potently than a TxA2 antagonist and stimulated the relation of intracellular secondary messengers greater than that of any TxA2 agonist, the effect of which was only partially inhibited by the TxA2 antagonist [121]. Wilson et al. found that pigs with hypercholesterolemia secondary to cholesterol diet had increased isoprostane levels independent of the TxA2 receptors [122]. It is possible that isoprostane mechanisms are tissue-specific, and further study is necessary to understand their biological effects in HF.

Multiple studies on F2-isoPs levels in CVD have been performed. Several case-control studies have found a clear association between elevated plasma and urinary F2-isoPs levels with the presence of coronary artery disease, compared to normal controls [123]. Roest et al. found that high levels of urinary F2-isoPs conferred a higher mortality risk secondary to coronary artery disease and stroke in postmenopausal women [55]. Another study by Leleiko et al. found plasma F2-isoPs levels greater than 124.5 pg/mL could predict poor outcomes in patients with acute coronary syndrome [53]. Several studies aimed to elucidate the role of F2-isoPs in patients with congestive HF. Mallat et al. found that pericardial levels of F2-isoPs were correlated with severity of HF and with the degree of ventricular dilatation, as seen on echocardiography. They also found that levels were correlated with left ventricular end-diastolic and end-systolic diameters, suggesting a potential role in stratifying the severity of HF [56].

Subsequently, Cracowski et al. found that urinary excretion of F2-isoPs was significantly higher in patients suffering from severe HF compared with age and sex matched controls. Urinary concentrations of F2-isoPs were higher in NYHA class IV compared to class II and III HF [124]. Radovanovic et al. also found higher urinary concentrations in NYHA class III and IV patients, as compared to NYHA I and control patients [125]. The receiver operating curve analysis identified a cut-off value of 0.84ng/mg, with sensitivity and specificity of above 95%. Additionally, echocardiographic indices of left ventricular remodeling such as the left ventricular end diastolic and end systolic diameter and volume were found to be directly related to the F2-isoPs levels.

These studies suggest that F2-isoPs may be used for HF prognostication as they correlate strongly with the functional severity of HF. Studies have elucidated the potential role of F2-isoPs as a potential biomarker for HF diagnosis, symptom severity, ejection fraction reduction and left ventricular remodeling. Urine and plasma samples are easily obtainable, and development of commercially available assays may translate to ease of measurement and clinical use. However, the role of F2-isoprostanes in long-term outcomes of HF, or its specific roles in HFpEF or HFrEF, is unclear and more studies may be required.

### 3.4. Protein Carbonyls

Protein carbonyl groups are general markers of OS and their associated diseases. Increased carbonyl levels have been seen in various disease states, such as Alzheimer’s disease, rheumatoid arthritis, diabetes, and chronic renal failure. Carbonyls are generated by direct oxidation of protein side chains or secondary oxidation from secondary reaction of the protein side chain with aldehydes from lipid peroxidation or with reactive carbonyl derivatives such as ketoamines, ketoaldehydes, deoxyozones. Assays for detection of protein CO groups involve derivatization of the carbonyl group with 2,4-dinitrophenylhydrazine (DNPH), which leads to formation of a stable dinitrophenyl (DNP) hydrazone product. This can be detected by various means, such as spectrophotometric assay, ELISA and one- or two-dimensional electrophoresis [126].

Elevated carbonyl levels in HF have been reported, albeit limited studies on it. Endothelin-1 is a vasoconstrictive peptide that mediates its effects via ROS generation, likely through ETA receptor activation and increased NAPH oxidase activity [127]. Increased ROS results in decarbonylation of multiple proteins, including annexin A1, an anti-inflammatory protein known to reduce cardiac inflammation [128], cardiac fibrosis and apoptosis and preserves cardiomyocyte survival in ischemic insult [129]. Endothelin itself plays a role in endothelin-mediated cell growth and survival of pulmonary artery smooth muscle cells [130], which are important events in pulmonary vascular remodeling. This leads to increased pulmonary vascular resistance, causing right ventricular strain and eventual right HF. Thus, deactivating protein carbonylation via endothelin antagonism is established as a therapeutic strategy in pulmonary hypertension [128,131].

The carbonylation of myofilament protein is another potential contributor to development of HF and is seen to occur in post-infarct mice. It decreases Ca^2+^ sensitivity and force production irrespective of myofilament phosphorylation status [132] This mechanism may also contribute to doxorubicin-induced cardiotoxicity and HF [133]. Hoshino et al. found that there are increased post-translational modifications including carbonylation of Lon protease homolog (LONP1), the most abundant mitochondrial AAA protease, in HF. This results in impaired electron transport chain, mitochondrial respiration deficiency and left ventricular contractile dysfunction [134]. Other proteins which have been found to undergo carbonylation in the setting of HF and OS include M type creatine kinase and alpha-cardiac actin, which result in impaired contractility [135].

Increased ROS-induced carbonyl modification of myocardial proteins occurs in the left ventricle of hamster models dilated and hypertrophic cardiomyopathy, with increased carbonyl level in cardiomyopathic hamsters compared to control animals. Carbonyl levels were inversely related to both succinyl-CoA:3-ketoacid-coenzyme A transferase 1 activity and ATP concentration, suggesting that the carbonylation of proteins in the myocardium may reduce ATP synthesis and contribute to poor ventricular function [136]. In a rat model, rats with DM1 and MI had worsened cardiac function compared to non-diabetic rats with MI, with higher carbonyl contents in cardiac tissue and isolated heart and significantly poorer residual LV systolic function and wet-to-dry weight ratios of the lungs [137].

Protein carbonylation has been found to be increased in HF patients compared to healthy controls [135]. A human study of hemodialysis patients found plasma protein carbonyl content and MDA to be elevated in patients with left ventricular hypertrophy compared to those with normal ventricular geometry. The incidence of eccentric left ventricular hypertrophy (LVH) significantly increased with protein carbonyl values, with the strong correlation suggesting that protein carbonyl levels may be good predictive markers of LVH development. Furthermore, carbonyl levels were independent predictors of higher LV end diastolic diameter and LV end-diastolic volume [58].

### 3.5. 8-Hydroxy-2′-deoxyguanosine (8-OHdG)

Apart from cellular proteins and lipids, OS can damage DNA and RNA in cells, making them potential biomarkers of total body OS. Examples of oxidative products include 8-Hydroxy-2′-deoxyguanosine (8-OhdG), and thymine glycol (TG). TG is a more specific marker of OS because thymidine is not incorporated into RNA. The mechanism behind DNA oxidative modification is unclear, and there is ongoing debate on whether the free nucleosides are oxidized prior to their incorporation into DNA molecules or there is direct oxidation of the DNA molecules. Regardless, oxidized nucleotides may lead to mismatch pairing and mutations, GC-TA mutations are related to cancer pathogenesis, including lung and breast cancer. To mitigate the harmful effects, cells have inherent repair mechanisms allowing for the excision and excretion of the oxidized nucleotide, leading to its eventual excretion in urine. There are several methods of measuring 8-OhdGs. High performance liquid chromatography (HPLC)-electrochemical detection is the most reliable method for measuring oxidative nucleotides (TG). ELISA and immunohistochemical analysis have been developed to measure TG in tissue samples.

Mitochondrial damage was found to be associated with HF [57,138]. OS can cause single-stranded DNA breaks, which activates the PARP-1 pathway for single strand DNA repair. An overactivation of PARP-1 depletes NAD^+^ and ATP pools leading to reduced mitochondrial respiration and cell death. Mitochondrial dysfunction is characterized by increased lipid peroxidation in the mitochondria, decreased mitochondrial DNA (mtDNA) copy number, fewer transcripts and reduced oxidative capacity [138]. OS can cause single-stranded DNA breaks, which activates the PARP-1 pathway for single strand DNA repair. mtDNA accumulates significantly greater levels of 8-OhdG compared to nuclear DNA. In fact, mtDNA has been suggested to be a target for ROS-mediated damage due to lack of histones for chromosomal protection, having a limited ability for DNA repair, and the mitochondria itself being a source of ROS with O_e_^−^ being retained in mitochondrial membranes [57].

The exact mechanism of mtDNA damage causing HF is not fully understood, but an enzymatic response to OS may be a possible biomarker in HF. 8-oxo-dGTPase, an enzyme that hydrolyzes 8-oxo-dGTP into 8-oxo-dGMP, has been shown to be increased in mitochondria isolated from failing hearts of post-MI mice along with OS, suggesting that an enzymatic response to OS may be a possible biomarker in HF [139]. Overexpression of peroxiredoxin-3 (Prx-3), mitochondrial antioxidant or mitochondrial transcription factor A (TFAM) could ameliorate the decline in mtDNA copy number in failing hearts [140].

8-OhdG was found to be significantly elevated in HF patients; correlated with LVEF, pulmonary wedge pressure, left ventricular end-diastolic volume index and BNP levels and correlated with NYHA classification [59]. It may also be affected by its etiology. Lipid peroxidation was measured in HF patients, and both markers were significantly higher compared to control subjects. The study grouped patients into three groups based on their etiology of their HF, ischemic cardiomyopathy, dilated cardiomyopathy and hypertensive cardiomyopathy. While all three groups showed a significant increase in both markers, both markers were seen to be highest in the hypertensive cardiomyopathy group [60].

HF patients with cardiovascular events had higher baseline 8-OhdG than HF patients without. Of those with cardiovascular events, those who had fatal events showed higher levels compared to nonfatal. 8-OhdG was also found to be an independent predictor of cardiac events in HF patients by Watanabe et al. [60]. Similarly, Suzuki et al. found serum 8-OhdG concentrations to be higher in HF patients than in controls and increasing with NYHA class. There was significant correlation between higher serum 8-OhdG and cardiac event rate [61]. A recent meta-analysis done by Di Minno et al., Watanabe et al. and 5 other studies found similar correlation between increasing NYHA class in HF and 8-OhdG levels [141].

RNA oxidation as a biomarker is of more recent interest. 7,8-dihydro-8-oxo-guanosine (8-oxoGuo) is an oxidized RNA nucleoside found to be correlated with several diseases, in particular neurodegenerative diseases and diabetes. Effects of oxidation on RNA transcripts are not entirely clear, though there is evidence to suggest that they produce a mixture of full-length and truncated proteins, mutated proteins, and cause stalling of the translation complex. There seems to be a difference in the amount of DNA and RNA oxidation depending on the disease. In diabetes, there is more RNA oxidation and has a different prognostic value compared to DNA oxidation.

### 3.6. Allantoin

In hominoid primates, UA is the final enzymatic product in the degradation pathway of purine nucleotides. It is formed when adenosine and guanosine are degraded to hypoxanthine and xanthine respectively. Hypoxanthine and xanthine are then oxidized by xanthine oxidase (XO) to form UA. UA can be further oxidized to allantoin by uricase in other mammals. This uniquely human “loss of function” and inability to further degrade UA to allantoin is hypothesized to have occurred during evolution secondary to a missense and frameshift mutation resulting in inactivation of the uricase gene. Humans therefore have higher levels of serum UA compared to other mammals. It is postulated that the loss of uricase conferred several evolutionary advantages such as a higher antioxidant capacity due to the higher levels of UA. UA functions as an antioxidant by binding with free radicals such as O_2_^−^ to form allantoin. Due to loss of the uricase gene in humans, allantoin can only be generated by free radical-mediated oxidation. As such, allantoin can possibly be used as a reliable biomarker for OS. Grootveld and Halliwell first demonstrated the possibility of quantifying allantoin levels within human plasma and synovial fluid using high performance liquid chromatography in 1987 [142]. Several in vivo studies have also demonstrated the relationship between OS and allantoin levels by generating OS through intense exercise.

Similar to humans, birds also lack uricase and cannot enzymatically convert UA to allantoin. Tsahar et al. successfully quantified plasma and ureteral allantoin levels in white-crowned sparrows before, during and after exercise in a hop or hover wheel and found a possible relationship between UA oxidation and free radical generation from exercise [143]. Mikami et al. evaluated the effect of exercise-induced OS in humans by correlating plasma and urinary allantoin levels before and after exhaustive static cycling and found a significant rise in both serum and urinary allantoin during and after exercise [144,145]. In another human study, Kandar et al. quantified the level of plasma allantoin before and after volunteers were subjected to a 10-min high-intensity run and found that plasma allantoin levels increased significantly immediately after exercise and returned to near baseline levels after 1 h of recovery [146]. In a more recent study, Haldar et al. evaluated the effects of an antioxidant polyphenol-rich diet on plasma allantoin levels in Chinese males and found that consumption of a polyphenol rich diet attenuated the rise in postprandial plasma allantoin and plasma allantoin to UA ratio, as compared to controls [147]. These experiments all suggest a relationship between the OS and allantoin levels.

As discussed earlier, OS plays an integral role in the development of CVD. Similar to in HF, OS and ROS have also been implicated contributors to atherosclerosis at least in part via effects of Ang II. A Brazilian cohort study (ELSA-Brazil) demonstrated that an elevated plasma allantoin level was associated with increased carotid intima-media thickness independent of established atherosclerotic risk factors [148]. These results not only suggest a strong relationship of OS with carotid atherosclerosis, but also demonstrate the utility of allantoin as a surrogate marker of OS. More specific to HF, Caussé et al. compared levels of aminothiols and purine degradation compounds in 75 HFrEF patients and 50 control patients, and specifically found that levels of purine degradation products, allantoin, UA, and allantoin to UA ratio were significantly higher in patients with HFrEF [149]. These results show a possible utility for plasma allantoin in the diagnosis of HF patients. Overall, allantoin appears to be a promising novel biomarker of OS in HF due to the development of reliable measurement techniques and a strong correlation between OS and plasma allantoin levels. More studies are needed however to elucidate the role of allantoin in HF prognosis, risk stratification and management.

### 3.7. Uric Acid

Uric acid (UA) is the end-product of purine metabolism, produced from the conversion of substrate xanthine by the enzyme xanthine oxidoreductase. It is a source of superoxide ions and contributes to OS in the system. It is reduced during catalysis of hypoxanthine to xanthine and xanthine to UA, and reoxidation involves electron transfer to oxygen, producing hydrogen peroxide and superoxide [150]. UA levels in HF may be more of a consequence of HF than a causative factor, as it is known to increase in hypoxic conditions and ischemia due to degradation of adenosine triphosphate via adenosine leads to increased substrate load for Xanthine oxidase. Hypoxia may worsen endothelial dysfunction via decreasing availability of NO and create a cycle where it promotes further ROS production and vessel dysfunction.

Higher UA levels in HF patients is correlated with disease severity. This increase in UA levels reflect xanthine oxidase activity independent of diuretic use and renal dysfunction. Prospective studies in patients with CHF showed that hyperuricemia is a marker of impaired oxidative metabolism and hyperinsulinemia, inflammatory cytokine activation and impaired vascular function. UA levels correlate to the NYHA classification and were significantly increased in symptomatic patients compared to asymptomatic patients, and had significant correlation with BNP and diuretic use [151]. It has also been shown to correlate with established prognostic factors in HF and may have additional prognostic value. High serum UA levels are a strong independent marker of impaired prognosis in moderate to severe HF, with a graded relationship with survival in CHF. In elderly patients with chronic HF, hyperuricemia was associated with higher cardiac events [152], and exercise intolerance [153].

UA may also predict HF development with raised serum UA associated with increased risk of HF in older men on antihypertensives. Some studies noted that more than 50% of CHF patients had elevated UA levels [62,63]. Hyperuricemia leads to worsening of diastolic function, reduce exercise performance, and cachexia [154,155,156]. Several longitudinal studies [64,157,158,159] and meta-analyses [65,160] indicated strong relationships between elevated UA levels and HF risk, severity and poor prognosis. According to the Framingham Offspring Cohort Study, the adjusted hazard ratio for HF incidence was 2.1 (95% CI: 1.04–4.22), in patients with high quartile of serum UA compared to those with low quartile [161]. In addition, Huang et al. reported the odds of HF incidence was increased by 19% (HR 1.19, 95% CI: 1.17–1.21), and the risk of all-cause mortality increased by 4% (HR 1.04, 95% CI: 1.02–1.06) [160]. According to the British Regional Heart Study, patients with high serum UA levels (>410 μmol/L) have an increased risk of HF [162]. Overall, these studies suggest UA is well established to be a strong potential OS biomarker for HF risk and adverse outcomes. Drugs that lower serum UA, such as xanthine oxidase inhibitors (allopurinol or febuxostat) have also shown benefits to HF patients [163,164].

## 4. Clinically Useful Biomarkers

### 4.1. Soluble NOX2-Derived Peptide

Recently, a systemic evaluation of NOX activity was proposed. An antibody was used to bind to the amino acid sequence (224–268) of the extramembrane portion of the NOX2. During cell activation, the expression of this amino-acid sequence was found to be reduced, concomitant with ROS burst. When the supernatant from the activated cells was analyzed, a peptide was recognized by the same antibody built against the amino-acid sequence (224–268) of the extramembrane portion of NOX2. This peptide was defined as a sNOX2-dp (soluble NOX2-derived peptide).

The sNOX2 peptide is measurable in both serum and plasma by an ELISA method that was developed to simplify the methodology. Further investigation showed that sNOX2-dp mostly represented the sum of the peptides released from blood cells, with circulating cells accounting for 90% of the released peptide, the remaining 10% assumed to be of endothelial cell origin. The use of sNOX2-dp is relatively new and information about its stability, metabolism and clearance is still lacking. ELISA studies have only been validated for plasma, serum, and cell supernatant samples in small populations, and not in large prospective studies. The small contribution to measurable sNOX2-dp from endothelial cells may make it less suitable for vascular studies. While limited clinical evidence is available for the use of sNOX-2-dp as a biomarker in HF, NOX2 has been implicated in other CVD including IHD and atrial fibrillation, which may precipitate future HF. We address how measuring NOX2 activity may be beneficial in such cases.

sNOX2-dp may be particularly useful for monitoring OS in HF because NOX expressed in cardiomyocytes has been shown to be the major source of ROS generation in pressure overload left ventricular hypertrophy in the pathogenesis of HF [165]. Similarly, Heymes et al. found increased NOX activity in end-stage failing myocardium compared to non-failing myocardium [166]. Interestingly, while Li et al. found an increase in expression of NOX subunits, Heymes et al. found overall level of oxidase subunit expression to be unaltered in failing compared to non-failing hearts. NOX2 has been suggested to be most responsible for generation of OS in development of HF, initiating various downstream pathways as discussed earlier.

The role of other NOX isoforms has been less studied and remains not well understood. Zhang et al. suggest that NOX4 may play a protective effect in chronic pressure overload, with NOX4 knockout mice developing worse left ventricular hypertrophy, contractile dysfunction and dilation while over-expression of NOX4 had the opposite effect [167]. However, another study showed that NOX4 had adverse effects in aortic constriction induced cardiac dysfunction [33]. ROS has been implicated in cardiac remodeling and development of HF after myocardial infarction. Multiple mechanisms have been proposed including those previously discussed. A major source of ROS in the post infarction setting is NOX2 [168]. A study found that NOX2 in the cardiomyocytes was responsible for the remodeling process, rather than NOX2 expression in the endothelium. By monitoring NOX2 activity specifically using sNOX-2-dp, one may be able to determine the degree of remodeling post-infarct. A study investigating the role of NOX2 in cardiac remodeling post-infarct in mice found NOX2 gene KO resulted in significantly less left ventricular cavity dilation and dysfunction compared to matched wild-type mice, suggesting that NOX2 contributes significantly to the pathogenesis of cardiac remodeling [169].

OS has been suggested to be a link between atrial fibrillation, tachycardia and atrial remodeling. A study found that NOX2 containing NADPH oxidase to be the main source of superoxide production in human atrial myocytes. There was an increase in NOX2 superoxide production in patients with atrial fibrillation compared to controls. Other contributors to OS include NOS and to a lesser extent, mitochondrial oxidases [170]. sNOX2-dp was elevated in persistent, paroxysmal atrial fibrillation and in permanent atrial fibrillation compared to healthy controls. The same study found sNOX2-dp to correlate strongly with urinary isoprostanes, suggesting that NOX2 may be responsible for isoprostane formation [171]. Violi et al. showed that NOX2 upregulation, measured in the form of sNOX2-dp was associated with increased risk of atrial fibrillation in patients with community acquired pneumonia [172].

Further studies are needed to understand the diagnostic and prognostic value of sNOX2-dp in HF. With increasing evidence showing that NOX2 is a major contributor to OS in HF, sNOX2-dp seems to be a potentially useful biomarker.

### 4.2. Myeloperoxidase

MPO is a heme-containing peroxidase enzyme found in neutrophils and macrophages. Its main role is to generate ROS in cells of the innate immune system to be used in degradation of bacteria after phagocytosis. MPO is beneficial for host defense, and a deficiency in MPO results in immune deficiency. MPO produces hypohalous acids, oxyacids which contain a halogen or pseudohalogen, and HOCl. Specific products to MPO include chlorotyrosine (3-Cl-Tyr), glutathione sulfonamide and chlorinated lipids. Increased MPO activity has been implicated in the pathogenesis of multiple diseases such as atherosclerosis, possibly as a manifestation of increased OS. MPO-derived ROS also reduce the bioavailability of NO, impairing vasodilation, and modifies high density lipoprotein, impairing its function in cholesterol efflux.

In HF, myeloperoxidase is associated with vascular dysfunction. Myeloperoxidase is thought to be electrostatically trapped by proteoglycans in the subendothelial glycocalyx. Its subsequent production of HOCl and other oxidants decreases the bioavailability of NO in vascular tissue. This impairs vasodilation, leading to hypoxia, ATP depletion, increased purine metabolism and accumulation of UA. MPO activity may also be induced by asymmetric dimethyl arginine (ADMA), which in turn is elevated by native or oxidized LDL. This thus creates a loop where high LDL level causes greater ADMA values which increases MPO activity and worsens vascular function. MPO and calprotectin are also involved in inflammatory responses in CVD.

The methods used to measure MPO activity have not been standardized and lack comparison studies between different methods of measurement. Myeloperoxidase itself can be measured by flow cytometry, immunohistochemistry, or cytochemical staining. Its product HOCl may also be measured by staining and spectroscopy. Some studies have also measured 3-Cl-Tyr, a specific product of MPO. Recently, ELISA kits are being more commonly used, and this has made testing more affordable. The variety of assays deployed in current studies which support or challenge the role of MPO in disease processes require validation. In general, measuring MPO and MPO-derived products remains expensive and time-consuming. These products are usually found in low concentrations that affect the measurement accuracy. Measurement of HDL, which was found to be a carrier of 3-Cl-Tyr, is possible but requires extensive preparation which limits clinical use. MPO measurement is also influenced by sample storage and time to analysis. Biomarkers of downstream effects of MPO activation are also possible, though they reflect more of endothelial microvascular involvement of OS. Calprotectin reflects neutrophil involvement, UA reflects tissue hypoxia, while arginine, ADMA and SDMA may reflect NO bioavailability.

Several studies have also investigated myeloperoxidase in chronic HF. Increased MPO levels were found in patients with HFpEF and correlated with UA levels. Diastolic dysfunction in HFpEF patients with E/e’ ratio of >14 associated with increasing UA levels, and more weakly with increasing MPO levels. UA was also significantly predictive of the combined endpoint of HF requiring hospitalization or all-cause death. Other markers found to be elevated in HFpEF were calprotectin, asymmetric dimethyl arginine, and symmetric dimethylarginine, while arginine was decreased [173]. Plasma MPO levels are raised in HFrEF, and in patients with chronic systolic HF compared to healthy controls and correlating with NYHA class and plasma BNP. These findings were independent of whether HF was of ischemic or non-ischemic etiology [174]. Ng et al. investigated use of MPO as a screening tool for HFrEF in combination with NT-proBNP [66]. The study screened 1360 individuals with 1331 having echocardiograph scans and plasma specimens. MPO individually was able to detect 27 out of 28 patients with undiagnosed HFrEF. Along with CRP, MPO was found to have an additive diagnostic value to BNP alone, and increased specificity for systolic HF. Furthermore, MPO had higher specificity than plasma BNP (74% compared to 41%). However, Reichlin et al. showed that MPO is not as useful as BNP in the diagnosis of acute HF [67]. In a study involving 667 patients with dyspnea presenting to the emergency department, MPO and BNP levels were measured. When compared to controls, it was found that MPO concentrations were similar in patients with acute HF compared to patients with noncardiac causes of dyspnea. MPO had less diagnostic value than BNP. Interestingly, when patients with acute HF in the study were followed up for 1 year, those with MPO levels above the lowest quartile saw a significant increase in 1-year mortality, after adjusting for other cardiovascular risk factors. This was in addition to the predictive factor from BNP, suggesting that MPO may be useful for risk management in acute HF patients. Further studies suggest MPO may provide prognostic value in HF. Tang et al. explored the relationship between MPO and cardiac dysfunction outcomes and found that an increase in plasma MPO levels correlated with restrictive diastolic stage, right ventricular systolic dysfunction and tricuspid regurgitation [68]. MPO was also predictive of long-term clinical outcomes of death, cardiac transplantation, or hospitalization due to HF.

In summary, while the role and evidence for MPO in HF is not as robust as in coronary heart disease, MPO is a potentially useful clinical diagnostic and prognostic biomarker for HF.

### 4.3. Advanced Glycation End-Products

Advanced glycation end-products (AGEs) are a group of compounds generated through nonenzymatic glycation and oxidation of proteins. AGEs may be formed through oxidative and non-oxidative pathways and may involve sugars or their degradation products. OS, though often involved, is not always necessary for AGE production. Briefly, OS induces lipid peroxidation and glycoxidation reactions, which lead to the formation of highly reactive and electrophilic compounds that attack free amino groups in proteins causing covalent modifications and resulting in the generation of AGEs [175].

In HF, AGEs are thought to bind to other AGEs and form additional cross-links between matrix proteins such as collagen, laminin and elastin. The cross-links reduce flexibility of matrix proteins that impair diastolic function. The activation of the receptor of AGE (RAGE), expressed at low levels in most tissues, may also contribute to HF. RAGE activation induces fibrosis via upregulation of transforming growth factor-beta and reduces contractility by altering calcium metabolism in cardiomyocytes. Transgenic mice with overexpressed human RAGE in the heart showed reduced systolic and diastolic intracellular calcium concentration. When expressed to AGEs, there was a significant delay in calcium uptake and prolonged repolarization leading to diastolic dysfunction [176]. Finally, AGEs may impair systolic function by enhancing the atherosclerotic process and development of CAD. AGEs may form crosslinks with LDL particles, making them more atherogenic by being less prone to reuptake by LDL receptors for clearance and increased uptake by macrophages to form foam cells [177].

Pentosidine, a subgroup of fluorescent AGEs, is commonly used to reflect the level of AGE and has been implicated in numerous diseases and ageing. Serum pentosidine has been associated with functional HF classification with pentosidine significantly higher in NYHA class III/IV compared to class I/II. Serum pentosidine was also elevated in patients with cardiac events compared to those without. Pentosidine was found to be an independent risk factor for cardiac events [178].

RAGE itself may also serve as a biomarker for HF. Serum RAGE includes both cleaved RAGE (cRAGE) and endogenously secreted RAGE (esRAGE). cRAGE is cleaved from cell surface membrane by metalloproteinases that are induced in HF. They do not neutralize AGEs, unlike esRAGE. A study suggested that both higher serum levels of cRAGE and lower serum levels of esRAGE correlate with severity of cardiac dysfunction, severity of symptoms and clinical outcomes in patients with HF [179]. A study measured serum soluble RAGE concentration in 160 patients and prospectively followed them up for a period of 872 days with endpoints of cardiac death or rehospitalization. Increased serum soluble RAGE concentration was correlated with increasing NYHA functional class and with cardiac events compared to those without cardiac events. Interestingly, soluble RAGE and serum pentosidine were found to be independent risk factors of cardiac events [69].

N-E-(carboxymethyl) lysine (CML) is another AGE under consideration as a biomarker. CML is generated by glyoxal, which may be formed by both lipid and sugar oxidation pathways. CML was shown to correlate with NYHA functional class [177]. Willemsen et al. measured plasma AGEs, CML, pentosidine and soluble form of RAGE in 580 hospitalized patients with HF when they were clinically stable with 18-month follow-up for the primary endpoint of death and HF admission. They found that CML and pentosidine levels were independently related to combined endpoint and HF hospitalization, and CML was independently related to increased mortality. This contrasted with soluble RAGE, which did not predict events [70].

AGEs have been implicated in both systolic and diastolic failure in diabetes. In HFpEF, a study found serum AGEs and CML to correlate with diastolic dysfunction in type 1 diabetes [180]. Increased tissue levels of AGE in diabetic HF were found to be independently associated with diastolic dysfunction and reduced exercise capacity compared to non-diabetic HF patients [181]. Soluble RAGE was also found to be higher in HFpEF [182]. In HFrEF, a study found serum AGE levels in type 1 diabetics to predict systolic impairment [183]. Biopsies of patients with HFrEF and HFpEF showed increased myocardial AGE deposition in patients with HFrEF but less so in patients with HFpEF. This was accompanied by increased collagen deposition in HFrEF [184].

### 4.4. Nitrotyrosine

Nitrotyrosine is a specific marker of nitrosative stress derived from the superoxide and nitric oxide reaction [185]. Protein tyrosine residues can be modified to form nitrotyrosine adducts by reactive nitrogen reactive species such as ONOO^−^ and nitrogen, though the exact mechanism is not well understood. Nitration of tyrosine involves the replacement of C3 hydrogen atom of the tyrosine aromatic ring with a nitro group (-NO_2_) and can occur to free tyrosine amino acids or to tyrosine in polypeptide chains.

Nitration can occur by several pathways in vivo, but always involves RNS and is usually a two-step process. The first step is the oxidation of tyrosine to form a tyrosine radical and the second step is the radical reaction between tyrosine radical and nitrogen dioxide. The initial oxidation usually involves ROS as the oxidizing agent, with superoxide and hydrogen peroxide both shown to be part of different tyrosine radical generation pathways. Nitrotyrosine was initially thought to reflect cellular damage by peroxynitrite since it was an intermediate in one of the pathways. However, the understanding of alternative pathways makes it nonspecific to ONOO^−^ production. The biological effect of nitration on proteins is variable, with possible change in structure and function [186].

Nitrosative stress plays an important role in the progression of chronic HF as discussed earlier. Similar to ROS, Reactive nitrogen species (RNS) leads to myocyte apoptosis, direct negative inotropic effects, and reduced bioavailability of nitric oxide (NO). RNS results in vasoconstriction of the coronary, pulmonary and peripheral vasculature. In patients with moderate to severe forms of chronic HF, nitrotyrosine causes depletion of nitric oxide through activation of myeloperoxidase (MPO). The mechanistic link of RNS, endothelial dysfunction and vascular inflammation in CHF, would likely contribute to progression of the disease severity. For example, nitrotyrosine formation on sarcoendoplasmic reticulum Ca^2+^-ATPase 2a (SERCA2a) was found to be significantly higher in cardiac tissue of patients with dilated cardiomyopathy compared with healthy controls [187].

Age-related cardiac decompensation is related to decreased rates of calcium transport mediated by the SERCA2a isoform of the sarcoplasmic reticulum, partly due to decreased SERCA2a expression but mostly due to decreased activity, as seen in rat studies. The additional loss of activity is a result of increased nitrotyrosine modification of the Ca-ATPase, which results in slower sequestration of cytosolic calcium, consequent prolonged muscle relaxation times, and dilated cardiomyopathy. Furthermore, generation of nitrotyrosine may activate MMPs in the development of HF. Homocysteine may decrease NO availability by generating nitrotyrosine, leading to activation of metalloproteinases. An increase in homocysteine in rats resulted in increased nitrotyrosine and subsequent continuous increase in MMP-2 activity at 4 and 8-weeks post administration. Removal of homocysteine did not decrease levels of nitrotyrosine nor decrease MMP-2 activity. In the absence of endothelial NO, MMP-2 was activated, and the corresponding tissue inhibitor was inactivated by the increasing nitrotyrosine to reduce LV load [188].

Nitrotyrosine may also contribute to HF in the setting of myocardial inflammation. A study of induced myocarditis in murine models showed that iNOS expression was increased in inflammatory macrophages and in distinct cardiomyocytes, which lead to increased nitrotyrosine. Notably, expression of iNOS and nitrotyrosine production was dependent on myocardial inflammation. This was shown to be regulated by interferon regulatory transcription factor 1. Mice defective for interferon regulatory transcription factor-1 after gene targeting was found to have no induction of iNOS and nitrotyrosine but developed myocarditis at prevalence and severity similar to controls [189].

A study found that iNOS expression correlates significantly with nitrotyrosine in idiopathic dilated cardiomyopathy. Patients with greater iNOS and nitrotyrosine levels had greater end diastolic and end systolic volume indices with similar LV end-diastolic pressure [190]. Hryniewicz et al. showed nitrotyrosine to be significantly increased in CHF when compared with normal subjects. Interestingly, nitrotyrosine was significantly elevated in non-ischemic CHF compared to ischemic CHF, non-diabetics, and subjects on statins [191]. No association was found with NYHA class and ejection fraction. However, another study showed nitrotyrosine levels were reported to be higher in NYHA III HF patients compared to NYHA II, I and controls while NO_2_ and total NO were higher in NYHA III compared to NYHA I and controls [71]. Nitrotyrosine correlated with MPO, TNF-alpha and NT-proBNP. Nitrotyrosine plasma levels are increased in moderate to severe HF patients in relation to systemic markers of inflammation.

### 4.5. Galectin-3

Galectin-3 is a carbohydrate-binding lectin that increases collagen production and cardiac fibroblast proliferation. Elevated levels of galectin-3 have been associated with macrophage infiltration, cardiac fibrosis, and cardiac hypertrophy, which contribute to progression of HF and poor cardiovascular outcomes. Measuring galectin-3 in conjunction with BNP/NT-proBNP and suppression of tumorigenicity 2 (ST2) may further enhance risk stratification to monitor and treat HF. Nguyen et al. explored mouse models of heart disease and in patients with cardiomyopathy, focusing on the context of galectin-3 [192]. Multi-fold increases in cardiac galectin-3 expression were observed in all the mouse models studied. There was also evidence of cardiac release of galectin-3 due to parallel changes in plasma and cardiac galectin-3 levels alongside the presence of the trans-galectin-3 gradient, showing the mediation of galectin-3 levels during cardiac inflammation and further supporting galectin-3′s value as a diagnostic marker of cardiac health.

The role of galectin-3 in cardiac biology is further explored in two studies on hypertrophied rat hearts and protein kinase C-(PKC) mediated cardiac fibrosis. Sharma et al. investigated the cardiac health of homozygous transgenic TGRmRen2-27 (Ren-2) rats [193]. By performing myocardial biopsy before the onset of hypertrophy, galectin-3 was identified to be significantly elevated in rats that develop HF. Furthermore, galectin-3 was observed to colocalize with activated myocardial macrophages, inducing fibroblast proliferation, cardiac dysfunction, and collagen deposition. Onset of ventricular dysfunction was observed after direct infusion of low doses of galectin-3 into the pericardial sac of Sprague-Dawley rats for 4 weeks, cementing the role of galectin-3 in HF and cardiac health. Song et al. investigated interactions between PKC-α and galectin-3, noting a strong correlation between galectin-3 levels and collagen I production [194]. In human biology, galectin-3 has been shown to have prognostic value in HF [72]. Another study by Medvedeva et al. in 2016 revealed evidence of elevated galectin-3 in patients with CHF as well as a positive correlation with OS and inflammation markers [195].

Finally, elevated galectin-3 levels were also observed in several large-scale population studies. Meijers et al. performed a detailed meta-analysis of 3 cohort studies (COACH, PRIDE and UMD H-23258, total of 902 patients) and reported significant improvement (42.6% continuous net improvement) in reclassification of patients with galectin-3 as an added independent variable [73]. With its biological relevance established and effects observed in patient cohorts, the FDA also approved galectin-3 as a novel biomarker for predicting adverse cardiac events in 2010.

### 4.6. Soluble Interleukin 1 Receptor-like 1

Another promising biomarker in cardiac biology is soluble interleukin 1 receptor-like 1 (ST2), or sST2, its soluble form. ST2 is a receptor for interleukin-33, a cytokine similar to IL-1. IL-33 has demonstrated cardioprotective effects in laboratory models, improving cardiac function while reducing the chance of onset of hypertrophy and fibrosis. These effects are strictly mediated by the interaction between IL-33 and ST2L, an isoform of ST2, whereas the interaction of IL-33 and the base form does not confer benefits. sST2 functions as a competitor receptor that reduces the effectiveness of its isoform ST2L when highly expressed. ST2 as a biomarker was first evaluated by Weinberg et al. using ELISA in 2003 [74]. Using the PRAISE-2 (n = 161) HF cohort as the subject population, baseline ST2 was found to be strongly correlated with BNP levels and proANP levels (*p* < 0.0001). In addition, change in ST2 for univariate models was a significant predictor of mortality. This finding is echoed in Ky et al.’s work with multi-institute studies of HF patients, which resulted in improving risk discrimination scores by 14.9% after incorporation of sST2 into the Seattle HF Model for prediction of adverse outcomes [75]. Bayes-Genis et al. confirmed the results, identifying both sST2 and NT-proBNP as significant risk factors in predicting mortality [76]. sST2 was also compared with galectin-3 for biomarker potential. While both biomarkers predicted all-cause mortality risk, sST2 was also able to predict cardiovascular mortality.

## 5. Surrogate Biomarkers

### 5.1. Antioxidants

High OS increases expression of antioxidant proteins, which can be measured and may reflect the degree of inciting OS Measuring antioxidant capacity may be done at multiple levels of gene expression. Firstly, transcription factors, for example nuclear factor erythroid 2-related factor 2 (NRF2) has been measured as a prognostic factor for several cancers, using Western blotting and q-RT-PCR in tumour biopsies. Secondly, reductive agents such as glutathione (GSH), thioredoxins, peroxiredoxins. Thirdly, enzymes in ROS degradation pathways, including catalase, glutathione peroxidase 1 (GPX-) and superoxide dismutase levels. These are generally easier to measure using antioxidant enzyme assays and stability of sample. Fourthly, the total antioxidative capacity (TAC), generally measured as the antioxidative activity of a sample against a generated radical, with measurement based on colorimetric methods on spectrophotometry. Further discusssion of specific molecules are discussed in this section.

Superoxide dismutase (SOD) is an enzyme that scavenges superoxide anion, thereby regulating the downstream reaction of superoxide with nitric oxide to form peroxynitrite anion, a reaction responsible for many pathological proceses [196]. SOD is a thought to correlate directly with the body’ level of OS. In STEMI patients post-PCI, higher SOD levels were a strong prognostic factor in predicting acute heHF, along with natriuretic peptide and nitrite/nitrates [77]. The study further showed that modifying the GRACE (Global Registry of Acute Coronary Events) risk stratification score with these parameters increased its predictive ability. Another more recent study showed that higher serum SOD activity was also poorly prognostic of long-term clinical outcomes in patients with non-ischemic dilated cardiomyopathy (NIDCM), after multivariate adjustments [78], presumably attributed to adaptive response to higher OS in the body. However, Li et al. found that decreasing SOD activity was associated with deterioration of LV geometry to overt HF with concentric and eccentric hypertrophy [197]. This may give a clue to the impact of OS in the onset of HF in patients with existing CVD and does not necessarily contradict the outcomes of previous studies.

### 5.2. Thiol-Containing Compounds

Thiol-containing compounds, including cysteine, glutathione, cysteamine and dihydrolipoic acid. These compounds act as a buffer to changes in oxidative/reductive stress. In high oxidative states, thiol-containing compounds are oxidized, increasing the disulfide pool and reducing OS. The reverse is true to counter reductive stress, mediated by reductases such as NADPH or NADH. Serum-free thiols have been studied in the context of HF, and were found to have a a strong negative correlation with OS in the form of reactive oxygen species (dROMs), a photometric assay that measures OS based on the Fenton reaction. Furthermore, thiol groups also had a significant negative correlation with NYHA classification and disease duration, suggesting that it may be used for diagnostic and monitoring purposes [198].

A study of serum-free thiols in stable chronic HF patients, found higher levels in younger patients with better renal function lower levels of NT-proBNP and PTH. This was also associated with favourable outcomes of decreased rehospitalisation and increased patient survival. Interestingly, this association was found to be insignificant after adjusting for cholesterol or established prognostic factors in HF, eGFR and NT-proBNP, suggesting a common pathophysiological pathway. A study on patients with ischemic disease without CAD found cystine, the oxidised form of cysteine and a marker of OS, was found to correlate negatively with the peak filling rate and positively with the left ventricular end-diastolic pressure, indicating that increased OS was associated with diastolic dysfunction. Interestingly, GSH was not found to correlate to diastolic dysfunction [79].

A commonly studied thiol containing compound is GSH, a tripeptide molecule that acts as a recycable source of cysteine to regulate OS. In an environment with high OS, GSH is oxidised by glutathione peroxidase instead of other protein thiols, protecting them from irreversible oxidation, which may lead to undesirable changes in protein structure and function. A study comparing GSH levels in HF patients and healthy controls found that GSH was decreased by 21% in NYHA class I patients with structural cardiac disease and by 40% in symptomatic patients of NYHA II to IV [80]. Samples were also taken from atrial appendages and found GSH levels in NYHA IV to be lower than NYHA I. In patients with CAD, the depletion in GSH was correlated to the left ventricle dysfunction. According to the functional NYHA class, significant depletion in blood GSH occurred before detectable elevation in blood soluble tumor necrosis factor receptor-1 (sTNFR1), a marker of symptomatic HF severity.

### 5.3. Paraoxonase-1

Paraoxonase-1 (PON1) is an antioxidizing enzyme that has an esterase and a paraoxonase activity. PON1 hydrolyses a wide variety of substrates including lipid peroxides. OS is thought to downregulate PON1 activity, secondary to systemic low-grade inflammation, which results in PON1 enzymatic activity exhaustion [199]. In HF, PON1 activity was significantly decreased, while the BNP and UA levels were significantly increased with the severity of disease [200] PON1 activity, specifically its arylesterase activity, was also shown to predict adverse HF [81] outcomes—low serum arylesterase activity was a significant predictor of developing future cardiovascular events, including MI and stroke [82]. Other than PON1 activity, the genetic marker of PON1 function has also been investigated as a marker of OS. PON1 Q192R gene variant is the strongest genetic biomarker of PON1 activity [201]. The PON1 Q192R variant showed a dose-dependent relationship to the severity of LVH and LV dysfunction.

### 5.4. Ceruloplasmin

Ceruloplasmin is a ferroxidase enzyme synthesized in the liver and the major copper-carrying protein in the blood. Its copper-dependent oxidase ability is most well-known, involving the oxidation of ferrous to ferric iron, allowing for transport of ferric iron via binding to transferrin and preventing it from participating in the generation of hydroxyl radicals. Ceruloplasmin was significantly increased in HF patients compared to controls in several studies. Andreasova et al. found that ceruloplasmin correlated significantly with NT-proBNP, NYHA classification and LVEF in HF patients and with NT-proBNP in controls [83]. Xu et al. also found increased ceruloplasmin levels correlating with the degree of HF [84]. While the ceruloplasmin levels were reportedly elevated in patients with advanced HF, it was accompanied by lower ferroxidase activity. This decrease in activity was explained to be secondary to oxidation of ceruloplasmin amino acids by peroxynitrite, produced in HF [202]. Studies have shown that peroxynitrite induces ceruloplasmin tyrosine nitration and cysteine thiol oxidation, which significantly reduces ferroxidase activity [85]. Reduced ferroxidase activity was associated with significant 2-year mortality, but ceruloplasmin itself was not associated with mortality in CHF.

Ceruloplasmin may also play a beneficial role in HF by inhibiting MPO generation, reducing hypochlorous acid production. A study comparing ceruloplasmin knockout rats and controls found that the deficiency of ceruloplasmin leads to increased oxidation of ascorbate due to myeloperoxidase activity. In terms of HF etiology, ceruloplasmin was observed to be high regardless of ischemic or non-ischemic aetiology but showed correlation with severity only in non-ischemic cardiomyopathy [203]. Serum ceruloplasmin was reported to be an independent predictor of survival in HF over a 5-year period [204]. The predictive effect was improved when used with BNP and other cardiac indices.

## 6. Molecular Therapeutic Targets

### 6.1. NADPH Oxidase Inhibition

This group of therapeutics inhibits the formation of superoxide ions generated by NADPH oxidase, found mainly in phagocytes and endothelial cells. Physiologically, superoxide generated by NADPH is used in the oxidation and deactivation of microbial pathogens. NADPH oxidase is a major source of superoxide ions; hence, the direct inhibition of NADPH may reduce OS and its adverse effects.

There are a wide range of compounds to inhibit NADPH with some having multiple actions. Diphenyleneiodonium is frequently used and inhibits a wide range of flavin-containing compounds. Diphenyleneiodonium is also known to inhibit NADH:ubiquinone oxidoreductase (also known as Complex I in the electron transport chain), NOS, xanthine oxidase and NADPH cytochrome p450 oxidoreductase. It then inhibits ROS generation from multiple sources, including mitochondrial and xanthine oxidase sources. Another NADPH oxidase inhibitor is apocynin, a new methoxy-substituted catechol that blocks the assembly of p47phox, the phagocyte NADPH oxidase organizer, into the membrane complex. This reduces superoxide production in rats and human vascular rings, increases NO production and improves endothelial function in ex vivo human arteries or veins. It has been reported that the administration in vivo of apocynin to deoxycorticosterone-acetate-salt hypertensive rats decreases both superoxide production and blood pressure [205]. In a rabbit study, apocynin was shown to have positive cardiac effects in post-infarct HF by reducing the decrease in cardiac sympathetic nerve terminal density and loss of function [206]. The study suggested that NADPH oxidase activation mediates cardiac sympathetic nerve terminal abnormalities in HF, and inhibition of NADPH oxidase could potentially improve heart function. However, apocynin required a high concentration to be effective.

Other studies have used chimeric peptide (gp91ds-tat). These proteins cross the cell membrane and inhibit p47phox associated with gp91phox. This was shown to significantly reduce Ang-II induced hypertension and vascular superoxide production. Gp91ds-tat improved cardiac contractile dysfunction and reduced infarct size in rat hearts subjected to ischemia-reperfusion injury when compared to controls [207]. This was thought to be explained by the inhibition of NADPH oxidase-induced superoxide release. The second part of the study showed that gp91ds-tat treated femoral vessels in rats. Increased NO release was noted by the end of perfusion, suggesting that gp91ds-tat mediates ischemia-reperfusion endothelial dysfunction.

Another compound used to inhibit NADPH is S17834, a benzo(b)pyran-4-one that was shown to inhibit NADPH oxidase, decreasing superoxide production and attenuating atherosclerotic lesions in apoprotein-E-deficient mice [203]. It is not known to scavenge superoxide or inhibit xanthine oxidase or eNOS. The exact mechanism is not well known, but S17834 is thought to also activate adenosine monophosphate-activated protein kinase (AMPK), which may explain its beneficial effects in animal models of atherosclerosis.

As explained previously, the most ideal marker of NADPH oxidase activity would be sNOX2-dp, an amino acid sequence that is released into systemic circulation on NADPH oxidase activity.

### 6.2. eNOS Uncoupling

BH4 has also been suggested to improve endothelial-dependent vasodilation. A study compared the increase in forearm blood flow as measured by plethysmography in CHF patients and healthy controls when acetylcholine intra-arterial administration was co-infused with BH4 and found that CHF patients showed a significant increase compared to controls [208]. Interestingly, BH4 co-infusion did not affect forearm blood flow when acetylcholine was replaced with sodium nitroprusside, nor did BH4 alter the baseline blood flow. Other than diabetes and hypertension, BH4 supplementation may have beneficial effects in diastolic dysfunction due to loss of estrogen. Rats with bilateral ovariectomy showed reduced cardiac BH4 when compared to healthy controls and was associated with impaired myocardial relaxation, augmented filling pressures, increased collagen deposition and thickened left ventricular walls [209]. The superoxide levels were also increased, while the NO level was decreased in rats with loss of estrogen. Chronic BH4 supplementation after bilateral ovariectomy improved diastolic function and attenuated left ventricular modelling while restoring myocardial nitric oxide release and preventing reactive oxygen species generation.

Chronic non-ischemic HF usually leads to atrial OS and electrophysiologic abnormalities by depletion of BH4 and NOS uncoupling. These electrical abnormalities resulting from non-ischemic HF may be mitigated by repletion of BH4, indicating a safe and effective maneuver to reduce the frequency of atrial arrhythmias during HF [210]. Studies show that acute and chronic administration of L-arginine improves vascular function in hypercholesterolemia and other forms of CVS disease. While the explanation of the L-arginine and NOS-reducing levels of OS through the reduction of NADPH levels and improving endothelial function through NO production is logical according to its metabolic pathway, L-arginine is found in high concentrations in cell and should not be the rate-limiting step in the reaction. A further increase in L-arginine concentration should not increase rate of reaction and improve endothelial function which has been reported in clinical studies. This contradiction is known as the L-arginine paradox, with baseline concentrations of L-arginine approximately 30 times higher than the Michaelis–Menten constant of the isolated purified eNOS in vitro. Multiple explanations have been suggested for the L-arginine paradox. One of the proposed mechanisms for improvement of endothelial function with L-arginine supplementation is the mechanism of L-arginine antagonizing (ADMA), an endogenous NOS inhibitor, which could potentially explain the paradox.

There is currently no known direct measurement of eNOS uncoupling activity. Measuring the products of NOS uncoupling, namely superoxide and NO, are alternatives but lack specificity and stability of products. Currently, studies on NOS uncoupling have used tissue homogenates and anti-NOS antibodies, with secondary antibodies added subsequently and visualized under chemiluminescence. Western blots have also been used to investigate the ratio of the NOS homodimer to monomer [211].

## 7. Conclusions

We reviewed current OS biomarkers linked to disease severity and their prognostic significance in HF. We also discussed potential biomarkers with therapeutic potential, and representative studies are illustrated in Figure 2. Representative studies of the prognostic value of OS biomarkers highlighting malondialdehyde (MDA), myeloperoxidase (MPO), nitrotyrosine and UA as key prognostic markers on all-cause mortality in HF. Although cardiac natriuretic peptides and troponins are well-established clinical biomarkers in HF, OS biomarkers are critical for risk stratification and long-term HF monitoring.

Even though many OS biomarkers are linked to disease severity, there is insufficient evidence for their specificity and clinical utility. Several lipid peroxidation markers (e.g., MDA and MPO) are promising predictors of mortality and play a significant role in OS-induced cardiomyopathy. UA, MDA, MPO, nitrotyrosine and oxLDL are currently the most promising OS markers, because they have the potential to predict major adverse cardiac events and all-cause mortality in HF. The discovery of new biomarkers (e.g., sNOX-2-dp, AGE, nitrotyrosine, ST2 and Galectin-3) reflects the complex interplay of various signaling components (inflammation, cardiac fibrosis, neurohumoral and matrix remodeling) related to the HFrEF and/or HFpEF phenotypes. Furthermore, oxidative post-translational modification of cardiac proteins, such as S-nitrosylation and S-glutathionylation of cysteine, promotes protein degradation and alters protein function irreversibly. Further enhancing the risk classification and monitoring precision might be a multi-marker strategy combining redox biomarkers with plasma BNP and serum cardiac troponin T. To thoroughly evaluate and establish their clinical value, additional study is warranted.

While OS has been shown to be closely intertwined with the development of HF, not all HF can be attributed to it. There is evidence to suggest that HF can occur in normal or even in reductive stress. A study collected serum from 54 HF patients for redox analysis including MDA, GSH, redox ratio (GSH/GSSG) and antioxidant enzyme activity [212]. Patients were grouped based on the calculated redox state (GSH/MDA ratio) into normal redox, hyper-oxidative and hyper-reductive groups. The results showed that most HF patients were in hyper-oxidative (42%) and normal redox states (41%), with a minority being in the hyper-reductive states (17%). Non-invasive echocardiography was used to determine cardiac function and remodeling, which found that 55% of hyper-oxidative patients had greater systolic dysfunction, while 62.5% of the hyper-reductive patients had higher diastolic function. Another limitation is the non-specificity of OS to HF. OS is implicated in many diseases other than HF such as CAD and peripheral vascular disease. Furthermore, many patients with HF have systemic metabolic disease with diabetes mellitus, hypertension and hyperlipidemia, all of which have been suggested to be a consequence of, or a cause of OS, hence making the interpretation of OS biomarkers for HF specifically a challenging task.

Classifying HF subtypes using biomarkers remain a clinical challenge. HFpEF and HFrEF have distinct phenotypes and pathogenetic etiology in terms of cytokines, extracellular matrix and inflammation. Both HFpEF and HFrEF have similar clinical characteristics, but mortality is higher in HFrEF than HFpEF. Managing HFpEF is more difficult, because it responds less well to treatments. Currently, only a few studies have evaluated potential biomarkers that could distinguish HFpEF and HFrEF, including C-reactive protein and troponin for HFrEF [213] and plasminogen activator inhibitor-1 and urinary albumin for HFpEF [214].

In the age of proteomics, lipidomics and metabolomics, and the advancement of techniques such as mass spectrometry, the generation of data for biomarker development is less becoming an obstacle, with many potential markers being discovered. This discovery-driven approach does not require previous knowledge of an existing molecule, rather it helps generate new hypothesis to explain available data [214]. Rather, the issues lie mainly in validation of biomarkers in clinical trials. There needs to be study designs that look into the clinical utility of biomarkers beyond the association of markers and disease states and severity [215]. One pitfall of OS biomarkers is their lack of specificity, and hence may not be diagnostically powerful enough on their own to provide a clinical diagnosis [215]. However, the value of such biomarkers becomes more evident when they suggest OS as a contributory underlying mechanism for a clinical phenotype. Subcategorizing clinically indistinguishable diseases with biochemical markers of OS may allow for further targeted therapy and better prognostication.

## References

[1] Bragazzi N.L., Zhong W., Shu J., Abu Much A., Lotan D., Grupper A., Younis A., Dai H. (2021). Burden of heart failure and underlying causes in 195 countries and territories from 1990 to 2017. Eur. J. Prev. Cardiol..

[2] Virani S.S., Alonso A., Aparicio H.J., Benjamin E.J., Bittencourt M.S., Callaway C.W., Carson A.P., Chamberlain A.M., Cheng S., Delling F.N. (2021). Heart Disease and Stroke Statistics-2021 Update: A Report From the American Heart Association. Circulation.

[3] Heidenreich P.A., Albert N.M., Allen L.A., Bluemke D.A., Butler J., Fonarow G.C., Ikonomidis J.S., Khavjou O., Konstam M.A., Maddox T.M. (2013). Forecasting the Impact of Heart Failure in the United States. Circ. Heart Fail..

[4] GBD 2015 Obesity Collaborators (2017). Health Effects of Overweight and Obesity in 195 Countries over 25 Years. N. Engl. J. Med..

[5] Sajedinejad S., Majdzadeh R., Vedadhir A., Tabatabaei M.G., Mohammad K. (2015). Maternal mortality: A cross-sectional study in global health. Glob. Health.

[6] Bahit M.C., Kochar A., Granger C.B. (2018). Post-Myocardial Infarction Heart Failure. JACC Heart Fail..

[7] Cook C., Cole G., Asaria P., Jabbour R., Francis D.P. (2014). The annual global economic burden of heart failure. Int. J. Cardiol..

[8] Heidenreich P.A., Bozkurt B., Aguilar D., Allen L.A., Byun J.J., Colvin M.M., Deswal A., Drazner M.H., Dunlay S.M., Evers L.R. (2022). AHA/ACC/HFSA Guideline for the Management of Heart Failure: A Report of the American College of Cardiology/American Heart Association Joint Committee on Clinical Practice Guidelines. Circulation.

[9] Bozkurt B., Coats A.J., Tsutsui H., Abdelhamid M., Adamopoulos S., Albert N., Anker S.D., Atherton J., Böhm M., Butler J. (2021). Universal definition and classification of heart failure: A report of the Heart Failure Society of America, Heart Failure Association of the European Society of Cardiology, Japanese Heart Failure Society and Writing Committee of the Universal Definition of Heart Failure: Endorsed by the Canadian Heart Failure Society, Heart Failure Association of India, Cardiac Society of Australia and New Zealand, and Chinese Heart Failure Association. Eur. J. Heart Fail..

[10] Berliner D., Bauersachs J. (2017). Current Drug Therapy in Chronic Heart Failure: The New Guidelines of the European Society of Cardiology (ESC). Korean Circ. J..

[11] Cao Y., Chen S., Liang Y., Wu T., Pang J., Liu S., Zhou P. (2018). Inhibition of hyperpolarization-activated cyclic nucleotide-gated channels by β-blocker carvedilol. Br. J. Pharmacol..

[12] Kaplinsky E., Mallarkey G. (2018). Cardiac myosin activators for heart failure therapy: Focus on omecamtiv mecarbil. Drugs Context.

[13] Lam C.S.P., Chandramouli C., Ahooja V., Verma S. (2019). SGLT-2 Inhibitors in Heart Failure: Current Management, Unmet Needs, and Therapeutic Prospects. J. Am. Heart Assoc..

[14] Wintrich J., Kindermann I., Ukena C., Selejan S., Werner C., Maack C., Laufs U., Tschöpe C., Anker S.D., Lam C.S.P. (2020). Therapeutic approaches in heart failure with preserved ejection fraction: Past, present, and future. Clin. Res. Cardiol. Off. J. Ger. Card. Soc..

[15] Abraham W.T., Fonarow G.C., Albert N.M., Stough W.G., Gheorghiade M., Greenberg B.H., O'Connor C.M., Sun J.L., Yancy C.W., Young J.B. (2008). Predictors of in-hospital mortality in patients hospitalized for heart failure: Insights from the Organized Program to Initiate Lifesaving Treatment in Hospitalized Patients with Heart Failure (OPTIMIZE-HF). J. Am. Coll. Cardiol..

[16] Loehr L.R., Rosamond W.D., Chang P.P., Folsom A.R., Chambless L.E. (2008). Heart failure incidence and survival (from the Atherosclerosis Risk in Communities study). Am. J. Cardiol..

[17] Senni M., Rodeheffer R.J., Tribouilloy C.M., Evans J.M., Jacobsen S.J., Bailey K.R., Redfield M.M. (1999). Use of echocardiography in the management of congestive heart failure in the community. J. Am. Coll. Cardiol..

[18] Modin D., Andersen D.M., Biering-Sorensen T. (2018). Echo and heart failure: When do people need an echo, and when do they need natriuretic peptides?. Echo. Res. Pract..

[19] Tribouilloy C., Rusinaru D., Mahjoub H., Goissen T., Lévy F., Peltier M. (2008). Impact of echocardiography in patients hospitalized for heart failure: A prospective observational study. Arch. Cardiovasc. Dis..

[20] Curtis J.P., Sokol S.I., Wang Y., Rathore S.S., Ko D.T., Jadbabaie F., Portnay E.L., Marshalko S.J., Radford M.J., Krumholz H.M. (2003). The association of left ventricular ejection fraction, mortality, and cause of death in stable outpatients with heart failure. J. Am. Coll. Cardiol..

[21] Shah A.M., Claggett B., Sweitzer N.K., Shah S.J., Anand I.S., Liu L., Pitt B., Pfeffer M.A., Solomon S.D. (2015). Prognostic Importance of Impaired Systolic Function in Heart Failure with Preserved Ejection Fraction and the Impact of Spironolactone. Circulation.

[22] Gouda P., Brown P., Rowe B.H., McAlister F.A., Ezekowitz J.A. (2016). Insights into the importance of the electrocardiogram in patients with acute heart failure. Eur. J. Heart Fail..

[23] Greig D., Austin P.C., Zhou L., Tu J.V., Pang P.S., Ross H.J., Lee D.S. (2014). Ischemic electrocardiographic abnormalities and prognosis in decompensated heart failure. Circ. Heart Fail..

[24] Park S.J., On Y.K., Byeon K., Kim J.S., Choi J.O., Choi D.J., Ryu K.H., Jeon E.S. (2013). Short- and long-term outcomes depending on electrical dyssynchrony markers in patients presenting with acute heart failure: Clinical implication of the first-degree atrioventricular block and QRS prolongation from the Korean Heart Failure registry. Am. Heart J..

[25] Shamim W., Francis D.P., Yousufuddin M., Varney S., Pieopli M.F., Anker S.D., Coats A.J. (1999). Intraventricular conduction delay: A prognostic marker in chronic heart failure. Int. J. Cardiol..

[26] Rankinen J., Haataja P., Lyytikäinen L.P., Huhtala H., Lehtimäki T., Kähönen M., Eskola M., Pérez-Riera A.R., Jula A., Niiranen T. (2020). Relation of intraventricular conduction delay to risk of new-onset heart failure and structural heart disease in the general population. Int. J. Cardiol. Heart Vasc..

[27] Salah K., Stienen S., Pinto Y.M., Eurlings L.W., Metra M., Bayes-Genis A., Verdiani V., Tijssen J.G.P., Kok W.E. (2019). Prognosis and NT-proBNP in heart failure patients with preserved versus reduced ejection fraction. Heart.

[28] Januzzi J.L., van Kimmenade R., Lainchbury J., Bayes-Genis A., Ordonez-Llanos J., Santalo-Bel M., Pinto Y.M., Richards M. (2006). NT-proBNP testing for diagnosis and short-term prognosis in acute destabilized heart failure: An international pooled analysis of 1256 patients: The International Collaborative of NT-proBNP Study. Eur. Heart J..

[29] Januzzi J.L., Camargo C.A., Anwaruddin S., Baggish A.L., Chen A.A., Krauser D.G., Tung R., Cameron R., Nagurney J.T., Chae C.U. (2005). The N-terminal Pro-BNP investigation of dyspnea in the emergency department (PRIDE) study. Am. J. Cardiol..

[30] Troughton R.W., Frampton C.M., Brunner-La Rocca H.P., Pfisterer M., Eurlings L.W., Erntell H., Persson H., O'Connor C.M., Moert D., Karlström P. (2014). Effect of B-type natriuretic peptide-guided treatment of chronic heart failure on total mortality and hospitalization: An individual patient meta-analysis. Eur. Heart J..

[31] Gaggin H.K., Januzzi J.L. (2013). Biomarkers and diagnostics in heart failure. Biochim. Biophys. Acta.

[32] de Jong J.W., Schoemaker R.G., de Jonge R., Bernocchi P., Keijzer E., Harrison R., Sharma H.S., Ceconi C. (2000). Enhanced Expression and Activity of Xanthine Oxidoreductase in the Failing Heart. J. Mol. Cell. Cardiol..

[33] Kuroda J., Ago T., Matsushima S., Zhai P., Schneider M.D., Sadoshima J. (2010). NADPH oxidase 4 (Nox4) is a major source of oxidative stress in the failing heart. Proc. Natl. Acad. Sci. USA.

[34] Yasunari K., Maeda K., Watanabe T., Nakamura M., Yoshikawa J., Asada A. (2004). Comparative effects of valsartan versus amlodipine on left ventricular mass and reactive oxygen species formation by monocytes in hypertensive patients with left ventricular hypertrophy. J. Am. Coll. Cardiol..

[35] Hirotani S., Otsu K., Nishida K., Higuchi Y., Morita T., Nakayama H., Yamaguchi O., Mano T., Matsumura Y., Ueno H. (2002). Involvement of Nuclear Factor-κB and Apoptosis Signal-Regulating Kinase 1 in G-Protein–Coupled Receptor Agonist–Induced Cardiomyocyte Hypertrophy. Circulation.

[36] Kim H.E., Dalal S.S., Young E., Legato M.J., Weisfeldt M.L., D'Armiento J. (2000). Disruption of the myocardial extracellular matrix leads to cardiac dysfunction. J. Clin. Investig..

[37] Li Y.Y., McTiernan C.F., Feldman A.M. (2000). Interplay of matrix metalloproteinases, tissue inhibitors of metalloproteinases and their regulators in cardiac matrix remodeling. Cardiovasc. Res..

[38] Spinale F.G., Coker M.L., Heung L.J., Bond B.R., Gunasinghe H.R., Etoh T., Goldberg A.T., Zellner J.L., Crumbley A.J. (2000). A Matrix Metalloproteinase Induction/Activation System Exists in the Human Left Ventricular Myocardium and Is Upregulated in Heart Failure. Circulation.

[39] Spinale F.G. (2002). Matrix metalloproteinases: Regulation and dysregulation in the failing heart. Circ. Res..

[40] Zhang H., Fan L., Liao H., Tu L., Zhang J., Xu D., Feng J. (2021). Correlations of cardiac function with inflammation, oxidative stress and anemia in patients with uremia. Exp. Ther. Med..

[41] Sundström J., Evans J.C., Benjamin E.J., Levy D., Larson M.G., Sawyer D.B., Siwik D.A., Colucci W.S., Sutherland P., Wilson P. (2004). Relations of Plasma Matrix Metalloproteinase-9 to Clinical Cardiovascular Risk Factors and Echocardiographic Left Ventricular Measures: The Framingham Heart Study. Circulation.

[42] Shults N.V., Melnyk O., Suzuki D.I., Suzuki Y.J. (2018). Redox Biology of Right-Sided Heart Failure. Antioxidants.

[43] Mikhael M., Makar C., Wissa A., Le T., Eghbali M., Umar S. (2019). Oxidative Stress and Its Implications in the Right Ventricular Remodeling Secondary to Pulmonary Hypertension. Front. Physiol..

[44] DeMarco V.G., Whaley-Connell A.T., Sowers J.R., Habibi J., Dellsperger K.C. (2010). Contribution of oxidative stress to pulmonary arterial hypertension. World J. Cardiol..

[45] Khoo N.K., Cantu-Medellin N., Devlin J.E., St Croix C.M., Watkins S.C., Fleming A.M., Champion H.C., Mason R.P., Freeman B.A., Kelley E.E. (2012). Obesity-induced tissue free radical generation: An in vivo immuno-spin trapping study. Free Radic. Biol. Med..

[46] Zelko I.N., Zhu J., Roman J. (2018). Role of SOD3 in silica-related lung fibrosis and pulmonary vascular remodeling. Respir. Res..

[47] Ghezzi P. (2020). Environmental risk factors and their footprints in vivo—A proposal for the classification of oxidative stress biomarkers. Redox Biol..

[48] Romuk E., Wojciechowska C., Jacheć W., Zemła-Woszek A., Momot A., Buczkowska M., Rozentryt P. (2019). Malondialdehyde and Uric Acid as Predictors of Adverse Outcome in Patients with Chronic Heart Failure. Oxidative Med. Cell. Longev..

[49] Radovanovic S., Savic-Radojevic A., Pljesa-Ercegovac M., Djukic T., Suvakov S., Krotin M., Simic D.V., Matic M., Radojicic Z., Pekmezovic T. (2012). Markers of oxidative damage and antioxidant enzyme activities as predictors of morbidity and mortality in patients with chronic heart failure. J. Card. Fail..

[50] Wojciechowska C., Jacheć W., Romuk E., Ciszek A., Bodnar P., Chwalba T., Waliczek M., Gąsior M., Rozentryt P. (2021). Serum Sulfhydryl Groups, Malondialdehyde, Uric Acid, and Bilirubin as Predictors of Adverse Outcome in Heart Failure Patients due to Ischemic or Nonischemic Cardiomyopathy. Oxidative Med. Cell. Longev..

[51] Wu A.H., Levy W.C., Welch K.B., Neuberg G.W., O'Connor C.M., Carson P.E., Miller A.B., Ghali J.K. (2013). Association Between Bilirubin and Mode of Death in Severe Systolic Heart Failure. Am. J. Cardiol..

[52] Okada A., Sugano Y., Nagai T., Honda Y., Iwakami N., Nakano H., Takashio S., Honda S., Asaumi Y., Aiba T. (2017). Usefulness of the Direct and/or Total Bilirubin to Predict Adverse Outcomes in Patients With Acute Decompensated Heart Failure. Am. J. Cardiol..

[53] Charach G., George J., Afek A., Wexler D., Sheps D., Keren G., Rubinstein A. (2009). Antibodies to oxidized LDL as predictors of morbidity and mortality in patients with chronic heart failure. J. Card. Fail..

[54] Jorde U.P., Colombo P.C., Ahuja K., Hudaihed A., Onat D., Diaz T., Hirsh D.S., Fisher E.A., Tseng C.H., Vittorio T.J. (2007). Exercise-Induced Increases in Oxidized Low-Density Lipoprotein Are Associated With Adverse Outcomes in Chronic Heart Failure. J. Card. Fail..

[55] Roest M., Voorbij H.A., Van der Schouw Y.T., Peeters P.H., Teerlink T., Scheffer P.G. (2008). High levels of urinary F2-isoprostanes predict cardiovascular mortality in postmenopausal women. J. Clin. Lipidol..

[56] Cracowski J.L., Tremel F., Marpeau C., Baguet J.P., Stanke-Labesque F., Mallion J.M., Bessard G. (2000). Increased formation of F(2)-isoprostanes in patients with severe heart failure. Heart (Br. Card. Soc.).

[57] Ide T., Tsutsui H., Kinugawa S., Utsumi H., Kang D., Hattori N., Uchida K., Arimura K.I., Egashira K., Takeshita A. (1999). Mitochondrial Electron Transport Complex I Is a Potential Source of Oxygen Free Radicals in the Failing Myocardium. Circ. Res..

[58] Dimitrijevic Z.M., Salinger Martinovic S.S., Nikolic V.N., Cvetkovic T.P. (2019). Protein Carbonyl Content Is a Predictive Biomarker of Eccentric Left Ventricular Hypertrophy in Hemodialysis Patients. Diagnostics.

[59] Kobayashi S., Susa T., Tanaka T., Wada Y., Okuda S., Doi M., Nao T., Yoshiga Y., Yamada J., Okamura T. (2011). Urinary 8-hydroxy-2′-deoxyguanosine reflects symptomatic status and severity of systolic dysfunction in patients with chronic heart failure. Eur. J. Heart Fail..

[60] Watanabe E., Matsuda N., Shiga T., Kajimoto K., Ajiro Y., Kawarai H., Kasanuki H., Kawana M. (2006). Significance of 8-Hydroxy-2′-Deoxyguanosine Levels in Patients With Idiopathic Dilated Cardiomyopathy. J. Card. Fail..

[61] Suzuki S., Shishido T., Ishino M., Katoh S., Sasaki T., Nishiyama S., Miyashita T., Miyamoto T., Nitobe J., Watanabe T. (2011). 8-Hydroxy-2′-deoxyguanosine is a prognostic mediator for cardiac event. Eur. J. Clin. Investig..

[62] Hamaguchi S., Furumoto T., Tsuchihashi-Makaya M., Goto K., Goto D., Yokota T., Kinugawa S., Yokoshiki H., Takeshita A., Tsutsui H. (2011). Hyperuricemia predicts adverse outcomes in patients with heart failure. Int. J. Cardiol..

[63] Thanassoulis G., Brophy J.M., Richard H., Pilote L. (2010). Gout, allopurinol use, and heart failure outcomes. Arch. Intern. Med..

[64] Wu A.H., Ghali J.K., Neuberg G.W., O’Connor C.M., Carson P.E., Levy W.C. (2010). Uric acid level and allopurinol use as risk markers of mortality and morbidity in systolic heart failure. Am. Heart J..

[65] Tamariz L., Harzand A., Palacio A., Verma S., Jones J., Hare J. (2011). Uric acid as a predictor of all-cause mortality in heart failure: A meta-analysis. Congest. Heart Fail..

[66] Ng L.L., Pathik B., Loke I.W., Squire I.B., Davies J.E. (2006). Myeloperoxidase and C-reactive protein augment the specificity of B-type natriuretic peptide in community screening for systolic heart failure. Am. Heart J..

[67] Reichlin T., Socrates T., Egli P., Potocki M., Breidthardt T., Arenja N., Meissner J., Noveanu M., Reiter M., Twerenbold R. (2010). Use of myeloperoxidase for risk stratification in acute heart failure. Clin. Chem..

[68] Tang W.H., Tong W., Troughton R.W., Martin M.G., Shrestha K., Borowski A., Jasper S., Hazen S.L., Klein A.L. (2007). Prognostic Value and Echocardiographic Determinants of Plasma Myeloperoxidase Levels in Chronic Heart Failure. J. Am. Coll. Cardiol..

[69] Koyama Y., Takeishi Y., Niizeki T., Suzuki S., Kitahara T., Sasaki T., Kubota I. (2008). Soluble Receptor for Advanced Glycation End Products (RAGE) is a Prognostic Factor for Heart Failure. J. Card. Fail..

[70] Willemsen S., Hartog J.W., van Veldhuisen D.J., van der Meer P., Roze J.F., Jaarsma T., Schalkwijk C., van der Horst I.C., Hillege H.L., Voors A.A. (2012). The role of advanced glycation end-products and their receptor on outcome in heart failure patients with preserved and reduced ejection fraction. Am. Heart J..

[71] Eleuteri E., Di Stefano A., Ricciardolo F.L., Magno F., Gnemmi I., Colombo M., Anzalone R., Cappello F., La Rocca G., Tarro Genta F. (2009). Increased nitrotyrosine plasma levels in relation to systemic markers of inflammation and myeloperoxidase in chronic heart failure. Int. J. Cardiol..

[72] Ahmad T., Fiuzat M., Neely B., Neely M., Pencina M., Kraus W., Zannad F., Whellan D., Donahue M., Piña I. (2014). Biomarkers of myocardial stress and fibrosis as predictors of mode of death in patients with chronic heart failure. JACC Heart Fail..

[73] Meijers W.C., Januzzi J.L., de Filippi C., Adourian A.S., Shah S.J., van Veldhuisen D.J., de Boer R.A. (2014). Elevated plasma galectin-3 is associated with near-term rehospitalization in heart failure: A pooled analysis of 3 clinical trials. Am. Heart J..

[74] Weinberg E.O., Shimpo M., Hurwitz S., Tominaga S., Rouleau J.L., Lee R.T. (2003). Identification of serum soluble ST2 receptor as a novel heart failure biomarker. Circulation.

[75] Ky B., French B., McCloskey K., Rame J.E., McIntosh E., Shahi P., Dries D.L., Tang W.H., Wu A.H., Fang J.C. (2011). High-sensitivity ST2 for prediction of adverse outcomes in chronic heart failure. Circ. Heart Fail..

[76] Hamaguchi A., de Antonio M., Vila J., Peñafiel J., Galán A., Barallat J., Zamora E., Urrutia A., Lupón J. (2014). Head-to-Head Comparison of 2 Myocardial Fibrosis Biomarkers for Long-Term Heart Failure Risk Stratification: ST2 Versus Galectin-3. J. Am. Coll. Cardiol..

[77] Parenica J., Kala P., Pavkova M.G., Tomandl J., Spinar J., Littnerova S., Jarkovsky J., Mebazaa A., Tomandlova M., Dastych M. (2016). Natriuretic peptides, nitrite/nitrate and superoxide dismutase have additional value on top of the GRACE score in prediction of one-year mortality and rehospitalisation for heart failure in STEMI patients—Multiple biomarkers prospective cohort study. Int. J. Cardiol..

[78] Romuk E., Jacheć W., Kozielska-Nowalany E., Birkner E., Zemła-Woszek A., Wojciechowska C. (2019). Superoxide dismutase activity as a predictor of adverse outcomes in patients with nonischemic dilated cardiomyopathy. Cell Stress Chaperones.

[79] Raad M., AlBadri A., Wei J., Mehta P.K., Maughan J., Gadh A., Thomson L., Jones D.P., Quyyumi A.A., Pepine C.J. (2020). Oxidative Stress Is Associated with Diastolic Dysfunction in Women with Ischemia with No Obstructive Coronary Artery Disease. J. Am. Heart Assoc..

[80] Thomson M.J., Frenneaux M.P., Kaski J.C. (2009). Antioxidant treatment for heart failure: Friend or foe?. QJM Int. J. Med..

[81] Hammadah M., Kalogeropoulos A.P., Georgiopoulou V.V., Weber M., Wu Y., Hazen S.L., Butler J., Tang W.H.W. (2017). High-density lipoprotein-associated paraoxonase-1 activity for prediction of adverse outcomes in outpatients with chronic heart failure. Eur. J. Heart Fail..

[82] Tang W.H.W., Hartiala J., Fan Y., Wu Y., Stewart A.F., Erdmann J., Kathiresan S., Roberts R., McPherson R., CARDIoGRAM Consortium (2012). Clinical and genetic association of serum paraoxonase and arylesterase activities with cardiovascular risk. Arterioscler. Thromb. Vasc. Biol..

[83] Andreasova T., Vondrakova D., Sedlackova L., Dvorak J., Taborsky L., Neuzil P., Malek F. (2018). Evaluation of Ceruloplasmin—A Potential Biomarker in Chronic Heart Failure. J. Clin. Exp. Cardiol..

[84] Xu Y., Lin H., Zhou Y., Cheng G., Xu G. (2013). Ceruloplasmin and the extent of heart failure in ischemic and nonischemic cardiomyopathy patients. Mediat. Inflamm..

[85] Cabassi A., Binno S.M., Tedeschi S., Ruzicka V., Dancelli S., Rocco R., Vicini V., Coghi P., Regolisti G., Montanari A. (2014). Low Serum Ferroxidase I Activity Is Associated with Mortality in Heart Failure and Related to Both Peroxynitrite-Induced Cysteine Oxidation and Tyrosine Nitration of Ceruloplasmin. Circ. Res..

[86] Galasko D., Montine T.J. (2010). Biomarkers of oxidative damage and inflammation in Alzheimer’s disease. Biomark. Med..

[87] Tkachenko H., Kurhaluk N., Grudniewska J., Andriichuk A. (2014). Tissue-specific responses of oxidative stress biomarkers and antioxidant defenses in rainbow trout Oncorhynchus mykiss during a vaccination against furunculosis. Fish Physiol. Biochem..

[88] Hecker M., Haurand M., Ullrich V., Diczfalusy U., Hammarström S. (1987). Products, kinetics, and substrate specificity of homogeneous thromboxane synthase from human platelets: Development of a novel enzyme assay. Arch. Biochem. Biophys..

[89] Folden D.V., Gupta A., Sharma A.C., Li S.Y., Saari J.T., Ren J. (2003). Malondialdehyde inhibits cardiac contractile function in ventricular myocytes via a p38 mitogen-activated protein kinase-dependent mechanism. Br. J. Pharmacol..

[90] Adams J.W., Sakata Y., Davis M.G., Sah V.P., Wang Y., Ligget S.B., Chien K.R., Brown J.H., Dorn G.W. (1998). Enhanced Galphaq signaling: A common pathway mediates cardiac hypertrophy and apoptotic heart failure. Proc. Natl. Acad. Sci. USA.

[91] Liao P., Wang S.-Q., Wang S., Zheng M., Zheng M., Zhang S.-J., Cheng H., Wang Y., Xiao R.-P. (2002). p38 Mitogen-activated protein kinase mediates a negative inotropic effect in cardiac myocytes. Circ. Res..

[92] Díaz-Vélez C.R., García-Castiñeiras S., Mendoza-Ramos E., Hernández-López E. (1996). Increased malon-dialdehyde in peripheral blood of patients with congestive heart failure. Am. Heart J..

[93] Polidori M.C., Savino K., Alunni G., Freddio M., Senin U., Sies H., Stahl W., Mecocci P. (2002). Plasma lipophilic antioxidants and malondialdehyde in congestive heart failure patients: Relationship to disease severity. Free. Radic. Biol. Med..

[94] Romuk E., Wojciechowska C., Jacheć W., Nowak J., Niedziela J., Malinowska-Borowska J., Głogowska-Gruszka A., Birkner E., Rozentryt P. (2019). Comparison of Oxidative Stress Parameters in Heart Failure Patients Depending on Ischaemic or Nonischaemic Aetiology. Oxidative Med. Cell. Longev..

[95] Tingberg E., Ohlin A.K., Gottsäter A., Ohlin H. (2006). Lipid peroxidation is not increased in heart failure patients on modern pharmacological therapy. Int. J. Cardiol..

[96] Sobotka P.A., Brottman M.D., Weitz Z., Birnbaum A.J., Skosey J.L., Zarling E.J. (1993). Elevated breath pentane in heart failure reduced by free radical scavenger. Free. Radic. Biol. Med..

[97] Yokokawa T., Sato T., Suzuki S., Oikawa M., Yoshihisa A., Kobayashi A., Yamaki T., Kunii H., Nakazato K., Suzuki H. (2017). Elevated exhaled acetone concentration in stage C heart failure patients with diabetes mellitus. BMC Cardiovasc. Disord..

[98] Samara M.A., Tang W.H., Cikach F., Gul Z., Tranchito L., Paschke K.M., Viterna J., Wu Y., Laskowski D., Dweik R.A. (2013). Single exhaled breath metabolomic analysis identifies unique breathprint in patients with acute decompensated heart failure. J. Am. Coll. Cardiol..

[99] Marcondes-Braga F.G., Gioli-Pereira L., Bernardez-Pereira S., Batista G.L., Mangini S., Issa V.S., Fernandes F., Bocci E.A., Ayub-Ferreira S.M., Mansur A.J. (2020). Exhaled breath acetone for predicting cardiac and overall mortality in chronic heart failure patients. ESC Heart Fail..

[100] Tranchito L., Albert C.L., Grove D., Gul Z., Cikach F., Dweik R., Tang W.H.W. (2017). Exhaled Acetone and Pentane in Acute Decompensated Heart Failure Correlate with Long Term Disease Free Survival. J. Card. Fail..

[101] Hokamaki J., Kawano H., Yoshimura M., Soejima H., Miyamoto S., Kajiwara I., Kojima S., Sakamoto T., Sugiyama S., Hirai N. (2004). Urinary biopyrrins levels are elevated in relation to severity of heart failure. J. Am. Coll. Cardiol..

[102] Shimomura H., Ogawa H., Takazoe K., Soejima H., Miyamoto S., Sakamoto T., Kawano H., Suefuji H., Nishikawa H., Arai H. (2002). Comparison of urinary biopyrrin levels in acute myocardial infarction (after reperfusion therapy) versus stable angina pectoris and their usefulness in predicting subsequent cardiac events. Am. J. Cardiol..

[103] Toyama K., Yamabe H., Uemura T., Nagayoshi Y., Morihisa K., Koyama J., Kanazawa H., Hoshiyama T., Ogawa H. (2013). Analysis of oxidative stress expressed by urinary level of 8-hydroxy-2′-deoxyguanosine and biopyrrin in atrial fibrillation: Effect of sinus rhythm restoration. Int. J. Cardiol..

[104] Alvarez A.M., Mukherjee D. (2011). Liver Abnormalities in Cardiac Diseases and Heart Failure. Int. J. Angiol..

[105] Nitti M., Furfaro A.L., Mann G.E. (2020). Heme Oxygenase Dependent Bilirubin Generation in Vascular Cells: A Role in Preventing Endothelial Dysfunction in Local Tissue Microenvironment?. Front. Physiol..

[106] Chintanaboina J., Haner M.S., Sethi A., Patel N., Tanyous W., Lalos A., Pancholy S. (2013). Serum bilirubin as a prognostic marker in patients with acute decompensated heart failure. Korean J. Intern. Med..

[107] Zheng H., Li Y., Xie N. (2014). Association of serum total bilirubin levels with diastolic dysfunction in heart failure with preserved ejection fraction. Biol. Res..

[108] Kudo K., Inoue T., Sonoda N., Ogawa Y., Inoguchi T. (2021). Relationship between serum bilirubin levels, urinary biopyrrin levels, and retinopathy in patients with diabetes. PLoS ONE.

[109] Kunii H., Ishikawa K., Yamaguchi T., Komatsu N., Ichihara T., Maruyama Y. (2009). Bilirubin and its oxidative metabolite biopyrrins in patients with acute myocardial infarction. Fukushima J. Med. Sci..

[110] Rodríguez-Sánchez E., Navarro-García J.A., González-Lafuente L., Aceves-Ripoll J., Vázquez-Sánchez S., Poveda J., Mercado-García E., Corbacho-Alonso N., Calvo-Bonacho E., Fernández-Velasco M. (2020). Oxidized Low-Density Lipoprotein Associates with Ventricular Stress in Young Adults and Triggers Intracellular Ca^2+^ Alterations in Adult Ventricular Cardiomyocytes. Antioxidants.

[111] Tang H.Y., Wang C.H., Ho H.Y., Wu P.T., Hung C.L., Huang C.Y., Wu P.R., Yeh Y.H., Cheng M.L. (2018). Lipidomics reveals accumulation of the oxidized cholesterol in erythrocytes of heart failure patients. Redox Biol..

[112] Rietzschel E.R., Langlois M., De Buyzere M.L., Segers P., De Bacquer D., Bekaert S., Cooman L., Van Oostveldt P., Verdonck P., De Backer G.G. (2008). Oxidized low-density lipoprotein cholesterol is associated with decreases in cardiac function independent of vascular alterations. Hypertension.

[113] Raikou V., Kardalinos V., Kyriaki D. (2018). Oxidized Low-Density Lipoprotein Serum Concentrations and Cardiovascular Morbidity in End Stage of Renal Disease. J. Cardiovasc. Dev. Dis..

[114] Yao Y.S., Li T.D., Zeng Z.H. (2020). Mechanisms underlying direct actions of hyperlipidemia on myocardium: An updated review. Lipids Health Dis..

[115] Tsutsui T., Tsutamoto T., Wada A., Maeda K., Mabuchi N., Hayashi M., Ohnishi M., Kinoshita M. (2002). Plasma oxidized low-density lipoprotein as a prognostic predictor in patients with chronic congestive heart failure. J. Am. Coll. Cardiol..

[116] Hodis H.N., Mack W.J., LaBree L., Mahrer P.R., Sevanian A., Liu C.R., Liu C.H., Hwang J., Selzer R.H., Azen S.P. (2002). Alpha-tocopherol supplementation in healthy individuals reduces low-density lipoprotein oxidation but not atherosclerosis: The Vitamin E Atherosclerosis Prevention Study (VEAPS). Circulation.

[117] Holder C., Adams A., McGahee E., Xia B., Blount B.C., Wang L. (2020). High-Throughput and Sensitive Analysis of Free and Total 8-Isoprostane in Urine with Isotope-Dilution Liquid Chromatography-Tandem Mass Spectrometry. ACS Omega.

[118] Sametz W., Grobuschek T., Hammer-Kogler S., Juan H., Wintersteiger R. (1999). Influence of isoprostanes on vasoconstrictor effects of noradrenaline and angiotensin II. Eur. J. Pharmacol..

[119] Yura T., Fukunaga M., Khan R., Nassar G.N., Badr K.F., Montero A. (1999). Free-radical–generated F2-isoprostane stimulates cell proliferation and endothelin-1 expression on endothelial cells. Kidney Int..

[120] Takahashi K., Nammour T.M., Fukunaga M., Ebert J., Morrow J.D., Roberts L.J., Hoover R.L., Badr K.F. (1992). Glomerular actions of a free radical-generated novel prostaglandin, 8-epi-prostaglandin F2 alpha, in the rat. Evidence for interaction with thromboxane A2 receptors. J. Clin. Investig..

[121] Fukunaga M., Makita N., Roberts L.J., Morrow J.D., Takahashi K., Badr K.F. (1993). Evidence for the existence of F2-isoprostane receptors on rat vascular smooth muscle cells. Am. J. Physiol.-Cell Physiol..

[122] Wilson S.H., Best P.J., Lerman L.O., Holmes D.R., Richardson D.M., Lerman A. (1999). Enhanced coronary vasoconstriction to oxidative stress product, 8-epi-prostaglandinF2α, in experimental hypercholesterolemia. Cardiovasc. Res..

[123] Zhang Z.J. (2013). Systematic review on the association between F2-isoprostanes and cardiovascular disease. Ann. Clin. Biochem..

[124] Mallat Z., Lebret I.P.M., Chatel D., Maclouf J., Tedgui A. (1998). Elevated levels of 8-iso-prostaglandin F2alpha in pericardial fluid of patients with heart failure: A potential role for in vivo oxidant stress in ventricular dilatation and progression to heart failure. Circulation.

[125] Radovanovic S., Krotin M., Simic D.V., Mimic-Oka J., Savic-Radojevic A., Pljesa-Ercegovac M., Matic M., Ninkovic N., Ivanovic B., Simic T. (2008). Markers of oxidative damage in chronic heart failure: Role in disease progression. Redox Rep..

[126] Dalle-Donne I., Rossi R., Giustarini D., Milzani A., Colombo R. (2003). Protein carbonyl groups as biomarkers of oxidative stress. Clin. Chim. Acta.

[127] Lund A.K., Peterson S.L., Timmins G.S., Walker M.K. (2005). Endothelin-1–Mediated Increase in Reactive Oxygen Species and NADPH Oxidase Activity in Hearts of Aryl Hydrocarbon Receptor (AhR) Null Mice. Toxicol. Sci..

[128] Wong C.M., Cheema A.K., Zhang L., Suzuki Y.J. (2008). Protein Carbonylation as a Novel Mechanism in Redox Signaling. Circ. Res..

[129] Qin C.X., Rosli S., Deo M., Cao N., Walsh J., Tate M., Alexander A.E., Donner D., Horlock D., Li R. (2019). Cardioprotective Actions of the Annexin-A1 N-Terminal Peptide, Ac2-26, Against Myocardial Infarction. Front. Pharmacol..

[130] Davie N., Haleen S.J., Upton P.D., Polak J.M., Yacoub M.H., Morrell N.W., Wharton J. (2002). ET(A) and ET(B) Receptors Modulate the Proliferation of Human Pulmonary Artery Smooth Muscle Cells. Am. J. Respir. Crit. Care Med..

[131] Love M.P., McMurray J.J. (1997). Endothelin in heart failure: A promising therapeutic target?. Heart.

[132] Balogh A., Santer D., Pásztor E.T., Tóth A., Czuriga D., Podesser B.K., Trescher K., Jaquet K., Erdodi F., Edes I. (2014). Myofilament protein carbonylation contributes to the contractile dysfunction in the infarcted LV region of mouse hearts. Cardiovasc. Res..

[133] Aryal B., Jeong J., Rao V.A. (2014). Doxorubicin-induced carbonylation and degradation of cardiac myosin binding protein C promote cardiotoxicity. Proc. Natl. Acad. Sci. USA.

[134] Hoshino A., Okawa Y., Ariyoshi M., Kaimoto S., Uchihashi M., Fukai K., Iwai-Kanai E., Matoba S. (2014). Oxidative Post-Translational Modifications Develop LONP1 Dysfunction in Pressure Overload Heart Failure. Circ. Heart Fail..

[135] Brioschi M., Polvani G., Fratto P., Parolari A., Agostoni P., Tremoli E., Banfi C. (2012). Redox proteomics identification of oxidatively modified myocardial proteins in human heart failure: Implications for protein function. PLoS ONE.

[136] Ichihara S., Suzuki Y., Chang J., Kuzuya K., Inoue C., Kitamura Y., Oikawa S. (2017). Involvement of oxidative modification of proteins related to ATP synthesis in the left ventricles of hamsters with cardiomyopathy. Sci. Rep..

[137] Dennis K.E., Hill S., Rose K.L., Sampson U.K., Hill M.F. (2013). Augmented cardiac formation of oxidatively-induced carbonylated proteins accompanies the increased functional severity of post-myocardial infarction heart failure in the setting of type 1 diabetes mellitus. Cardiovasc. Pathol..

[138] Ide T., Tsutsui H., Hayashidani S., Kang D., Suematsu N., Nakamura K., Utsumi H., Hamasaki N., Takeshita A. (2001). Mitochondrial DNA Damage and Dysfunction Associated With Oxidative Stress in Failing Hearts After Myocardial Infarction. Circ. Res..

[139] Tsutsui H., Ide T., Shiomi T., Kang D., Hayashidani S., Suematsu N., Wen J., Utsumi H., Hamasaki N., Takeshita A. (2001). 8-Oxo-dGTPase, Which Prevents Oxidative Stress-Induced DNA Damage, Increases in the Mitochondria From Failing Hearts. Circulation.

[140] Matsushima S., Ide T., Yamato M., Matsusaka H., Hattori F., Ikeuchi M., Kubota T., Sunagawa K., Hasegawa Y., Kurihara T. (2006). Overexpression of mitochondrial peroxiredoxin-3 prevents left ventricular remodeling and failure after myocardial infarction in mice. Circulation.

[141] Di Minno A., Turnu L., Porro B., Squellerio I., Cavalca V., Tremoli E., Di Minno M.N. (2017). 8-Hydroxy-2-deoxyguanosine levels and heart failure: A systematic review and meta-analysis of the literature. Nutr. Metab. Cardiovasc. Dis..

[142] Grootveld M., Halliwell B. (1987). Measurement of allantoin and uric acid in human body fluids. A potential index of free-radical reactions in vivo?. Biochem. J..

[143] Tsahar E., Arad Z., Izhaki I., Guglielmo C.G. (2006). The relationship between uric acid and its oxidative product allantoin: A potential indicator for the evaluation of oxidative stress in birds. J. Comp. Physiol. B.

[144] Mikami T., Kita K., Tomita S., Qu G.J., Tasaki Y., Ito A. (2000). Is allantoin in serum and urine a useful indicator of exercise-induced oxidative stress in humans?. Free Radic. Res..

[145] Mikami T., Yoshino Y., Ito A. (2000). Does a relationship exist between the urate pool in the body and lipid peroxidation during exercise?. Free Radic. Res..

[146] Kanďár R., Štramová X., Drábková P., Křenková J. (2014). A monitoring of allantoin, uric acid, and malondialdehyde levels in plasma and erythrocytes after ten minutes of running activity. Physiol. Res..

[147] Haldar S., Pakkiri L.S., Lim J., Chia S.C., Ponnalagu S., Drum C.L., Henry C.J. (2018). Reductions in Postprandial Plasma Allantoin Concentrations With Increasing Doses of Polyphenol Rich Curry Intake—A Randomized Crossover Trial. Front. Physiol..

[148] Santana M.S., Nascimento K.P., Lotufo P.A., Benseãor I.M., Meotti F.C. (2018). Allantoin as an independent marker associated with carotid intima-media thickness in subclinical atherosclerosis. Braz. J. Med. Biol. Res..

[149] Caussé E., Fournier P., Roncalli J., Salvayre R., Galinier M. (2017). Serum allantoin and aminothiols as biomarkers of chronic heart failure. Acta Cardiol..

[150] Zimmet J.M., Hare J.M. (2006). Nitroso–Redox Interactions in the Cardiovascular System. Circulation.

[151] Khan A., Shah M.H., Khan S., Shamim U., Arshad S. (2017). Serum Uric Acid level in the severity of Congestive Heart Failure (CHF). Pak. J. Med. Sci..

[152] Niizeki T., Takeishi Y., Arimoto T., Okuyama H., Nozaki N., Hirono O., Tsunoda Y., Watanabe T., Nitobe J., Miyashita T. (2006). Hyperuricemia associated with high cardiac event rates in the elderly with chronic heart failure. J. Cardiol..

[153] Jankowska E.A., Ponikowska B., Majda J., Zymlinski R., Trzaska M., Reczuch K., Borodulin-Nadzieja L., Banasiak W., Ponikowski P. (2007). Hyperuricaemia predicts poor outcome in patients with mild to moderate chronic heart failure. Int. J. Cardiol..

[154] Doehner W., Rauchhaus M., Florea V.G., Sharma R., Bolger A.P., Davos C.H., Coats A.J., Anker S.D. (2001). Uric acid in cachectic and noncachectic patients with chronic heart failure: Relationship to leg vascular resistance. Am. Heart J..

[155] Leyva F., Anker S., Swan J.W., Godsland I.F., Wingrove C.S., Chua T.P., Stevenson J.C., Coats A.J. (1997). Serum uric acid as an index of impaired oxidative metabolism in chronic heart failure. Eur. Heart J..

[156] Amin A., Vakilian F., Maleki M. (2011). Serum uric acid levels correlate with filling pressures in systolic heart failure. Congest. Heart Fail..

[157] Anker S.D., Doehner W., Rauchhaus M., Sharma R., Francis D., Knosalla C., Davos C.H., Cicoira M., Shamim W., Kemp M. (2003). Uric acid and survival in chronic heart failure: Validation and application in metabolic, functional, and hemodynamic staging. Circulation.

[158] Ekundayo O.J., Dell'Italia L.J., Sanders P.W., Arnett D., Aban I., Love T.E., Filippatos G., Anker S.D., Lloyd-Jones D.M., Bakris G. (2010). Association between hyperuricemia and incident heart failure among older adults: A propensity-matched study. Int. J. Cardiol..

[159] Holme I., Aastveit A.H., Hammar N., Jungner I., Walldius G. (2009). Uric acid and risk of myocardial infarction, stroke and congestive heart failure in 417,734 men and women in the Apolipoprotein MOrtality RISk study (AMORIS). J. Intern. Med..

[160] Huang H., Huang B., Li Y., Huang Y., Li J., Yao H., Jing X., Chen J., Wang J. (2014). Uric acid and risk of heart failure: A systematic review and meta-analysis. Eur. J. Heart Fail..

[161] Krishnan E. (2009). Hyperuricemia and incident heart failure. Circ. Heart Fail..

[162] Wannamethee S.G., Papacosta O., Lennon L., Whincup P.H. (2018). Serum uric acid as a potential marker for heart failure risk in men on antihypertensive treatment: The British Regional Heart Study. Int. J. Cardiol..

[163] Doehner W., Schoene N., Rauchhaus M., Leyva-Leon F., Pavitt D.V., Reaveley D.A., Schuler G., Coats A.J., Anker S.D., Hambrecht R. (2002). Effects of xanthine oxidase inhibition with allopurinol on endothelial function and peripheral blood flow in hyperuricemic patients with chronic heart failure: Results from 2 placebo-controlled studies. Circulation.

[164] Xu X., Hu X., Lu Z., Zhang P., Zhao L., Wessale J.L., Bache R.J., Chen Y. (2008). Xanthine oxidase inhibition with febuxostat attenuates systolic overload-induced left ventricular hypertrophy and dysfunction in mice. J. Card. Fail..

[165] Li J.-M., Gall N.P., Grieve D.J., Chen M., Shah A.M. (2002). Activation of NADPH Oxidase during Progression of Cardiac Hypertrophy to Failure. Hypertension.

[166] Heymes C., Bendall J.K., Ratajczak P., Cave A.C., Samuel J.L., Hasenfuss G., Shah A.M. (2003). Increased myocardial NADPH oxidase activity in human heart failure. J. Am. Coll. Cardiol..

[167] Zhang M., Brewer A.C., Schröder K., Santos C.X., Grieve D.J., Wang M., Anilkumar N., Yu B., Dong X., Walker S.J. (2010). NADPH oxidase-4 mediates protection against chronic load-induced stress in mouse hearts by enhancing angiogenesis. Proc. Natl. Acad. Sci. USA.

[168] Grieve D.J., Byrne J.A., Cave A.C., Shah A.M. (2004). Role of Oxidative Stress in Cardiac Remodelling after Myocardial Infarction. Heart Lung Circ..

[169] Looi Y.H., Grieve D.J., Siva A., Walker S.J., Anilkumar N., Cave A.C., Marber M., Monaghan M.J., Shah A.M. (2008). Involvement of Nox2 NADPH Oxidase in Adverse Cardiac Remodeling after Myocardial Infarction. Hypertension.

[170] Kim Y.M., Guzik T.J., Zhang Y.H., Zhang M.H., Kattach H., Ratnatunga C., Pillai R., Channon K.M., Casadei B. (2005). A Myocardial Nox2 Containing NAD(P)H Oxidase Contributes to Oxidative Stress in Human Atrial Fibrillation. Circ. Res..

[171] Cangemi R., Celestini A., Calvieri C., Carnevale R., Pastori D., Nocella C., Vicario T., Pignatelli P., Violi F. (2012). Different behaviour of NOX2 activation in patients with paroxysmal/persistent or permanent atrial fibrillation. Heart.

[172] Violi F., Carnevale R., Calvieri C., Nocella C., Falcone M., Farcomeni A., Taliani G., Cangemi R., SIXTUS Study Group (2015). Nox2 up-regulation is associated with an enhanced risk of atrial fibrillation in patients with pneumonia. Thorax.

[173] Hage C., Michaëlsson E., Kull B., Miliotis T., Svedlund S., Linde C., Donal E., Daubert J.C., Gan L.M., Lund L.H. (2020). Myeloperoxidase and related biomarkers are suggestive footprints of endothelial microvascular inflammation in HFpEF patients. ESC Heart Fail..

[174] Tang W.H., Brennan M.L., Philip K., Tong W., Mann S., Van Lente F., Hazen S.L. (2006). Plasma myeloperoxidase levels in patients with chronic heart failure. Am. J. Cardiol..

[175] Vistoli G., De Maddis D., Cipak A., Zarkovic N., Carini M., Aldini G. (2013). Advanced glycoxidation and lipoxidation end products (AGEs and ALEs): An overview of their mechanisms of formation. Free. Radic. Res..

[176] Periasamy M., Huke S. (2001). SERCA Pump Level is a Critical Determinant of Ca2+Homeostasis and Cardiac Contractility. J. Mol. Cell. Cardiol..

[177] Hartog J.W.L., Voors A.A., Bakker S.J., Smit A.J., van Veldhuisen D.J. (2007). Advanced glycation end-products (AGEs) and heart failure: Pathophysiology and clinical implications. Eur. J. Heart Fail..

[178] Koyama Y., Takeishi Y., Arimoto T., Niizeki T., Shishido T., Takahashi H., Nozaki N., Hirono O., Tsunoda Y., Nitobe J. (2007). High Serum Level of Pentosidine, an Advanced Glycation End Product (AGE), is a Risk Factor of Patients with Heart Failure. J. Card. Fail..

[179] Neeper M., Schmidt A.M., Brett J., Yan S.D., Wang F., Pan Y.C., Elliston K., Stern D., Shaw A. (1992). Cloning and expression of a cell surface receptor for advanced glycosylation end products of proteins. J. Biol. Chem..

[180] Berg T.J., Snorgaard O., Faber J., Torjesen P.A., Hildebrandt P., Mehlsen J., Hanssen K.F. (1999). Serum levels of advanced glycation end products are associated with left ventricular diastolic function in patients with type 1 diabetes. Diabetes Care.

[181] Willemsen S., Hartog J.W., Hummel Y.M., van Ruijven M.H., van der Horst I.C., van Veldhuisen D.J., Voors A.A. (2011). Tissue advanced glycation end products are associated with diastolic function and aerobic exercise capacity in diabetic heart failure patients. Eur. J. Heart Fail..

[182] Raposeiras-Roubín S., Rodiño-Janeiro B.K., Grigorian-Shamagian L., Moure-González M., Seoane-Blanco A., Varela-Román A., Almenar-Bonet L., Alvarez E., González-Juanatey J.R. (2011). Relation of Soluble Receptor for Advanced Glycation End Products to Predict Mortality in Patients With Chronic Heart Failure Independently of Seattle Heart Failure Score. Am. J. Cardiol..

[183] Steine K., Larsen J.R., Stugaard M., Berg T.J., Brekke M., Dahl-Jørgensen K. (2007). LV systolic impairment in patients with asymptomatic coronary heart disease and type 1 diabetes is related to coronary atherosclerosis, glycaemic control and advanced glycation endproducts. Eur. J. Heart Fail..

[184] van Heerebeek L., Hamdani N., Handoko M.L., Falcao-Pires I., Musters R.J., Kupreishvili K., Ijsselmuiden A.J., Schalkwijk C.G., Bronzwaer J.G., Diamant M. (2008). Diastolic stiffness of the failing diabetic heart: Importance of fibrosis, advanced glycation end products, and myocyte resting tension. Circulation.

[185] Tomin T., Schittmayer M., Honeder S., Heininger C., Birner-Gruenberger R. (2019). Irreversible oxidative post-translational modifications in heart disease. Expert Rev. Proteom..

[186] Frijhoff J., Winyard P.G., Zarkovic N., Davies S.S., Stocker R., Cheng D., Knight A.R., Taylor E.L., Oettrich J., Ruskovska T. (2015). Clinical Relevance of Biomarkers of Oxidative Stress. Antioxid. Redox Signal..

[187] Knyushko T.V., Sharov V.S., Williams T.D., Schöneich C., Bigelow D.J. (2005). 3-Nitrotyrosine modification of SERCA2a in the aging heart: A distinct signature of the cellular redox environment. Biochemistry.

[188] Sood H.S., Cox M.J., Tyagi S.C. (2002). Generation of Nitrotyrosine Precedes Activation of Metalloproteinase in Myocardium of Hyperhomocysteinemic Rats. Antioxid. Redox Signal..

[189] Bachmaier K., Neu N., Pummerer C., Duncan G.S., Mak T.W., Matsuyama T., Penninger J.M. (1997). iNOS expression and nitrotyrosine formation in the myocardium in response to inflammation is controlled by the interferon regulatory transcription factor 1. Circulation.

[190] Vanderheyden M., Bartunek J., Knaapen M., Kockx M., De Bruyne B., Goethals M. (2004). Hemodynamic effects of inducible nitric oxide synthase and nitrotyrosine generation in heart failure. J. Heart Lung Transplant..

[191] Hryniewicz K., Yasskiy A., O’Donnell L., Zheng H., Katz S.D. (2004). Clinical correlates of nitrotyrosine as a marker of oxidative stress in heart failure. J. Card. Fail..

[192] Nguyen M.N., Su Y., Vizi D., Fang L., Ellims A.H., Zhao W.-B., Kiriazis H., Gao X.-M., Sadoshima J., Taylor A.J. (2018). Mechanisms responsible for increased circulating levels of galectin-3 in cardiomyopathy and heart failure. Sci. Rep..

[193] Sharma U.C., Pokharel S., van Brakel T.J., van Berlo J.H., Cleutjens J.P., Schroen B., André S., Crijns H.J., Gabius H.J., Maessen J. (2004). Galectin-3 marks activated macrophages in failure-prone hypertrophied hearts and contributes to cardiac dysfunction. Circulation.

[194] Song X., Qian X., Shen M., Jiang R., Wagner M.B., Ding G., Chen G., Shen B. (2015). Protein kinase C promotes cardiac fibrosis and heart failure by modulating galectin-3 expression. Biochim. Biophys. Acta.

[195] Medvedeva E.A., Berezin I.I., Surkova E.A., Yaranov D.M., Shchukin Y.V. (2016). Galectin-3 in patients with chronic heart failure: Association with oxidative stress, inflammation, renal dysfunction and prognosis. Minerva Cardioangiol..

[196] Fukai T., Folz R.J., Landmesser U., Harrison D.G. (2002). Extracellular superoxide dismutase and cardiovascular disease. Cardiovasc. Res..

[197] Li X., Lin Y., Wang S., Zhou S., Ju J., Wang X., Chen Y., Xia M. (2020). Extracellular Superoxide Dismutase Is Associated with Left Ventricular Geometry and Heart Failure in Patients with Cardiovascular Disease. J. Am. Heart Assoc..

[198] Yordanova M.G. (2020). Research of Oxidative Stress and Serum Thiols as a Criterion for the Antioxidant Barrier in Patients with Heart Failure (NYHA FC III-IV). Online J. Cardiol. Res. Rep..

[199] Kotur-Stevuljevic J., Spasic S., Jelic-Ivanovic Z., Spasojevic-Kalimanovska V., Stefanovic A., Vujovic A., Memon L., Kalimanovska-Ostric D. (2008). PON1 status is influenced by oxidative stress and inflammation in coronary heart disease patients. Clin. Biochem..

[200] Eren E., Ellidag E.Y., Aydin O., Kucukseymen O.G., Aslan S., Yilmaz N. (2015). The relationship between HDL-associated PON1 activity, oxidative stress and brain natriuretic peptide in NYHA functional class ii-iv heart failure patients. Biomed. Res..

[201] Dounousi E., Bouba I., Spoto B., Pappas K., Tripepi G., Georgiou I., Tselepis A., Elisaf M., Tsakiris D., Zoccali C. (2016). A Genetic Biomarker of Oxidative Stress, the Paraoxonase-1 Q192R Gene Variant, Associates with Cardiomyopathy in CKD: A Longitudinal Study. Oxidative Med. Cell. Longev..

[202] Shiva S., Wang X., Ringwood L.A., Xu X., Yuditskaya S., Annavajjhala V., Miyajima H., Hogg N., Harris Z.L., Gladwin M.T. (2006). Ceruloplasmin is a NO oxidase and nitrite synthase that determines endocrine NO homeostasis. Nat. Chem. Biol..

[203] Cayatte A.J., Rupin A., Oliver-Krasinski J., Maitland K., Sansilvestri-Morel P., Boussard M.F., Wierzbicki M., Verbeuren T.J., Cohen R.A. (2001). S17834, a New Inhibitor of Cell Adhesion and Atherosclerosis That Targets NADPH Oxidase. Arterioscler. Thromb. Vasc. Biol..

[204] Dadu R.T., Dodge R., Nambi V., Virani S.S., Hoogeveen R.C., Smith N.L., Chen F., Pankow J.S., Guild C., Tang W.H. (2013). Ceruloplasmin and Heart Failure in the Atherosclerosis Risk in Communities Study. Circ. Heart Fail..

[205] Chen Q.Z., Han W.Q., Chen J., Zhu D.L., Gao P.J. (2013). Anti-stiffness effect of apocynin in deoxycorticosterone acetate-salt hypertensive rats via inhibition of oxidative stress. Hypertens. Res..

[206] Wang K., Zhu Z.F., Chi R.F., Li Q., Yang Z.J., Jie X., Hu X.L., Han X.B., Wang J.P., Li B. (2019). The NADPH oxidase inhibitor apocynin improves cardiac sympathetic nerve terminal innervation and function in heart failure. Exp. Physiol..

[207] Young L., Walker S. (2014). Gp91ds-tat, a selective NADPH oxidase peptide inhibitor, increases blood nitric oxide bioavailability in hind limb ischemia and reperfusion (667.7). FASEB J..

[208] Setoguchi S., Hirooka Y., Eshima K., Shimokawa H., Takeshita A. (2002). Tetrahydrobiopterin Improves Impaired Endothelium-Dependent Forearm Vasodilation in Patients with Heart Failure. J. Cardiovasc. Pharmacol..

[209] Jessup J.A., Zhang L., Presley T.D., Kim-Shapiro D.B., Wang H., Chen A.F., Groban L. (2011). Tetrahydro-biopterin Restores Diastolic Function and Attenuates Superoxide Production in Ovariectomized mRen2.Lewis Rats. Endocrinology.

[210] Carnicer R., Crabtree M.J., Sivakumaran V., Casadei B., Kass D.A. (2013). Nitric oxide synthases in heart failure. Antioxid. Redox Signal..

[211] Bendall J.K., Alp N.J., Warrick N., Cai S., Adlam D., Rockett K., Yokoyama M., Kawashima S., Channon K.M. (2005). Stoichiometric Relationships Between Endothelial Tetrahydrobiopterin, Endothelial NO Synthase (eNOS) Activity, and eNOS Coupling in Vivo. Circ. Res..

[212] Sairam T., Patel A.N., Subrahmanian M., Gopalan R., Pogwizd S.M., Ramalingam S., Sankaran R., Rajasekaran N.S. (2018). Evidence for a hyper-reductive redox in a sub-set of heart failure patients. J. Transl. Med..

[213] de Boer R.A., Nayor M., deFilippi C.R., Enserro D., Bhambhani V., Kizer J.R., Blaha M.J., Brouwers F.P., Cushman M., Lima J.A. (2018). Association of Cardiovascular Biomarkers With Incident Heart Failure With Preserved and Reduced Ejection Fraction. JAMA Cardiol..

[214] Khan S.S., Shah S.J., Klyachko E., Baldridge A.S., Eren M., Place A.T., Aviv A., Puterman E., Lloyd-Jones D.M., Heiman M. (2017). A null mutation in SERPINE1 protects against biological aging in humans. Sci. Adv..

[215] McDermott J.E., Wang J., Mitchell H., Webb-Robertson B.J., Hafen R., Ramey J., Rodland K.D. (2013). Challenges in biomarker discovery: Combining expert insights with statistical analysis of complex omics data. Expert Opin. Med. Diagn..

